# Development and performance evaluation of a clinical metagenomics approach for identifying pathogens in the whole blood from patients with undifferentiated fever

**DOI:** 10.3389/fcimb.2025.1667422

**Published:** 2025-09-15

**Authors:** Jan Slunečko, Rok Kogoj, Samo Zakotnik, Alen Suljič, Nataša Knap, Martin Bosilj, Franc Strle, Tatjana Avšič-Županc, Petra Bogovič, Miša Korva

**Affiliations:** ^1^ Institute of Microbiology and Immunology, Faculty of Medicine, University of Ljubljana, Ljubljana, Slovenia; ^2^ Department of Infectious Diseases, University Medical Center Ljubljana, Ljubljana, Slovenia

**Keywords:** mNGS, clinical metagenomics, molecular diagnostics, universal pathogen detection, enhanced RNA virus detection

## Abstract

**Introduction:**

Blood culture is the cornerstone of microbiological diagnostics for patients with acute undifferentiated fever and no obvious localization of infection; however, up to 50% of cases remain undiagnosed. Infections caused by arboviruses, fastidious or even uncultivable bacteria, or parasites often go undiagnosed without the use of target-specific molecular methods. These are typically performed in a stepwise manner, increasing cost and delaying results. Metagenomic next-generation sequencing (mNGS) has recently gained recognition as a potential universal pathogen detection tool for such cases. Our study aimed to develop a streamlined mNGS workflow for simultaneous detection of intracellular and cell-free pathogens within a single sequencing library.

**Methods:**

Total nucleic acid was isolated separately from 200 EDTA blood samples. The plasma isolate was processed with DNase, followed by the depletion of host ribosomal and messenger RNA, reverse transcription, and sequence-independent single primer amplification (SISPA). The whole blood isolate was only reverse transcribed, with no other pre-processing manipulation. Finally, the two fractions were combined prior to library preparation and sequencing using either Oxford Nanopore Technologies or Illumina. Following established bioinformatics analysis, we developed a mathematical ranking approach (ClinSeq score) that enabled quick identification of relevant pathogens in approximately one hour.

**Results:**

The mNGS workflow reached 79.5% (159/200) overall sensitivity. For bacteria the sensitivity was 88.6% (70/79), DNA viruses, 66.7% (10/15) and for RNA viruses 73.8% (76/103). Pathogen detections by individual sequencing methods showed overall sensitivity of Illumina and ONT to be 80.0% (76/95) and 79.1% (83/105) respectively. The ClinSeq score correctly highlighted the pathogen in 126/200 (63.0%) samples effectively with a Cohen’s kappa (*κ*) agreement of 0.61 with manual analysis.

**Conclusion:**

Developed comprehensive mNGS workflow detects a wide range of pathogens in patients with acute undifferentiated fever. The unified workflow improves sensitivity for intracellular bacteria and RNA viruses, reduces time, cost and complexity by eliminating the need for separate library preparations, enabling faster turnaround suitable for clinical settings. The ClinSeq score effectively differentiates true pathogen signals from background noise, reducing false positives and manual interpretation time. Overall, the workflow demonstrates flexible, and efficient pathogen detection, supporting its potential for clinical diagnostics and improved patient management.

## 1 Introduction

Clinical metagenomic next-generation sequencing (mNGS) is emerging as a powerful diagnostic tool for the diagnostics of undifferentiated fever, which can have numerous infectious or non-infectious causes (Fu et al., 2022). Contemporary microbiological laboratory diagnostic methods are able to identify a causative agent in up to 50% of patients with acute undifferentiated fever with no obvious localization of infection (Bleeker-Rovers et al., 2007). While up to 15% of these cases can be resolved by blood cultures (Foong et al., 2022), they are often limited by collection procedure issues i.e. one set instead of the recommended two or three, under- or overfilling, contamination (Doern et al., 2019; Khare et al., 2020), antibiotic treatment prior to blood drawing (Fabre et al., 2020; Lin et al., 2023; Zeng et al., 2025), and its inherent inability to detect bacteria with special atmosphere (microaerophilic) growth requirements, slow growing bacteria, intracellular bacteria, or biochemically inactive bacteria (Fenollar and Raoult, 2007; Lamy et al., 2016; Zeng et al., 2025). Parallelly, PCR tests are limited by target specificity, pathogen nucleic acid integrity and *a priori* suspicion of the causative agent (Batool and Galloway-Peña, 2023). In such cases, several recent studies have demonstrated the added value of mNGS (Hong et al., 2020; Hu et al., 2021; Lu et al., 2022; Qi et al., 2023; Zhou et al., 2023). Metagenomic NGS has been used effectively to uncover viral, bacterial and parasitic etiologies in patients with febrile illness (Jerome et al., 2019; Kandathil et al., 2024; Ashraf et al., 2025; Lai et al., 2025). However, the variety of protocols in terms of sample preparation, nucleic acid extraction strategies, sequencing depth, and data interpretation pipelines makes direct comparison difficult and the general application of mNGS to clinical diagnostics challenging (Korzeniewski et al., 2015; Marra et al., 2024). Most of the protocols developed so far focus exclusively on targeting viral pathogens, particularly in cases of febrile illness in returning travelers, where arboviruses are commonly suspected to be the cause of fever (Jerome et al., 2019; Kandathil et al., 2024). Conversely, studies that rely solely on DNA sequencing are inherently limited in their ability to detect RNA viruses. While some studies have attempted to address this issue and demonstrated the feasibility of sequencing both DNA and RNA pathogens simultaneously (Carbo et al., 2020; Lopez-Labrador et al., 2024), other studies have shown that separating the sample into two sub-samples, which are then processed separately, enhances pathogen detection (Lewandowska et al., 2017; Miller et al., 2019; Arroyo Mühr et al., 2021; Cebriá-Mendoza et al., 2021; Atkinson et al., 2023; Buddle et al., 2024; Lopez-Labrador et al., 2024). Although some studies have investigated a wide variety of clinical sample types, plasma is generally favored due to its lower human background (Lewandowska et al., 2017; Cheng et al., 2019; Niles et al., 2023; Lopez-Labrador et al., 2024). However, this approach reduces the sensitivity in detecting intracellular pathogens, such as *Babesia* sp., *Ehrlichia* sp. and *Plasmodium* sp (Cunha et al., 2008; Wass et al., 2018; Erdem et al., 2024). As preparing DNA/RNA in parallel and duplicating NGS libraries increases the costs of mNGS, sample pooling was investigated (Teufel and Sobetzko, 2022). However, this is only possible when large batches of clinical samples are processed at once, which rarely occur in diagnostics. Conversely, the bioinformatic processing of sequencing data is equally challenging. This involves setting the correct thresholds for the number of reads needed to distinguish between background noise, contamination and pathogens. The outcome of taxonomic classification is highly sensitive to variability across bioinformatic pipelines (Bosilj et al., 2024). The choice of reference databases, classification algorithms, quality filtering, and downstream processing steps can all influence the number of reads assigned to a given taxon. These factors are compounded by upstream variables, including the biological background of the sample, laboratory procedures and sequencing technology. Consequently, standardized pipelines are required, and the interpretation of processed data must be as unbiased as possible to establish reporting criteria capable of distinguishing true positives from false ones (Bosilj et al., 2024).

The aim of the study was to develop a streamlined mNGS workflow comprising of a wet-lab protocol, and dry-lab analysis for the detection of fastidious and uncultivable pathogens, enabling the simultaneous detection of cell-free and intracellular pathogens, within a single sequencing library. To facilitate laborious result analysis and interpretation, a data driven mathematical ranking approach (ClinSeq score) has been developed.

## 2 Materials and methods

### 2.1 Sample selection

A total of 200 EDTA blood samples collected from patients with acute undifferentiated fever (body temperature > 38.3 °C; > 3 days) and no obvious localization of infection were included in the validation of the developed mNGS workflow. As an additional inclusion criterion, the etiology of the disease had to be confirmed with validated molecular tests during routine diagnostics. Information regarding the molecular tests employed in this study can be found in **Supplementary Material** (**Supplementary Table S1**). The list of pathogens in individual samples was: fastidious bacteria (*Anaplasma phagocytophilum*, *Bartonella quintana*, *Coxiella burnettii*, *Francisella tularensis*, *Leptospira* sp., *Neoehrlichia mikurensis*, *Capnocytophaga canimorsus)*, viruses with RNA genome (chikungunya virus (CHIKV), dengue virus (DENV), Dobrava virus (DOBV), Puumala virus (PUUV), tick-borne encephalitis virus (TBEV), yellow fever virus (YFV) and Zika virus (ZIKV)), viruses with DNA genome (cytomegalovirus (CMV), Epstein–Barr virus (EBV) and parvovirus B19 (PB19)) and parasites (*Babesia* sp. and *Plasmodium falciparum*). Detailed sample data can be found in the **Supplementary Material** (**Supplementary Table S2**).

### 2.2 Molecular detection assays

All molecular detections assays, except for EBV and CMV, are qualitative assays and the results are reported as positive or negative (**Supplementary Table S1**). Only for the purpose of this study, to compare relative abundance of pathogens, we retrieved cycle threshold (Ct) values from each assay. For EBV and CMV Ct values and quantitative values, expressed in international units per ml (IU/ml), were retrieved.

### 2.3 Sample processing

A total of 600 μl of blood sample per patient was retrieved from storage at −80 °C. Total nucleic acid (NA) was isolated separately from 300 μl of EDTA whole blood and from 300 μl of plasma using the TANBead OptiPure Viral Auto Plate kit (TANbead inc., Taoyuan City, Taiwan) on the Maelstrom 9600 instrument (TANbead inc., Taoyuan City, Taiwan). In both cases, the elution volume was 60 μl. Following NA isolation, only the plasma isolates underwent treatment with the TURBO DNA-*free*™ Kit (Thermo Fisher Scientific, Waltham, MA, USA). DNA content was measured using the Qubit dsDNA High Sensitivity Assay (Thermo Fisher Scientific, Waltham, MA, USA) on a Qubit 3.0 fluorometer (Thermo Fisher Scientific, Waltham, MA, USA). Depletion of host ribosomal RNA (rRNA) and globin messenger RNA (mRNA) was achieved using the QIAseq FastSelect -rRNA/Globin kit (QIAGEN, Hilden, Germany) at a 1:10 dilution of each QIAseq reagent (Ramachandran et al., 2022).

### 2.4 Random primer amplification

For complementary DNA (cDNA) generation and subsequent random primer amplification, we employed the sequence-independent, single-primer amplification (SISPA) protocol. Total NA isolated from whole blood was processed directly with the SISPA-A protocol without any pretreatment. DNase- and QIAseq-treated NA isolated from plasma was processed with both SISPA-A and SISPA-B (Reyes and Kim, 1991; Chrzastek et al., 2017; Moore et al., 2020). Briefly, double-stranded complementary DNA (ds-cDNA) was generated using a SISPA-A primer (5′-GTTTCCCAGTCACGATC-N9-3′). Amplification of the resulting ds-cDNA was performed by utilizing the barcoded SISPA-A primer and its complementary SISPA-B primer (5′-GTTTCCCAGTCACGATC-3′). The necessary purification steps were carried out using AMPure XP magnetic beads (Beckman Coulter, Brea, CA, USA) at a 1:1 ratio, according to the manufacturer’s instructions. Purified SISPA-A from whole blood and purified SISPA-B from plasma were then mixed at a 1:1 ratio prior to NGS library generation. The detailed protocol for both (plasma and whole blood) is available in the **Supplementary Material** (**Supplementary Data S3**).

### 2.5 NGS library preparation

Libraries were prepared using either the NexteraXT DNA library preparation kit (Illumina, San Diego, CA, USA) or Native Barcoding Kit v14 (Oxford Nanopore Technologies, Oxford, UK), according to the manufacturer’s instructions. The necessary purification steps were carried out using AMPure XP magnetic beads (Beckman Coulter, Brea, CA, USA). Final pool concentration was measured using the Qubit dsDNA High Sensitivity Assay (Thermo Fisher Scientific, Waltham, MA, USA) on a Qubit 3.0 instrument (Thermo Fisher Scientific, Waltham, MA, USA), and the fragment size was analyzed using the Agilent High Sensitivity DNA Kit on the Bioanalyzer 2100 (both Agilent Technologies, Santa Clara, CA, USA).

### 2.6 Sequencing

Samples prepared with the NexteraXT DNA library preparation kit (Illumina, San Diego, CA, USA) were sequenced on a NextSeq 500/550 HighOutput Kit v2.5 (300 cycles) cartridge (Illumina, San Diego, CA, USA) with a target of 5 million reads per sample. Samples prepared with Native Barcoding Kit v14 (Oxford Nanopore Technologies, Oxford, UK) were analyzed in batches of 20 samples on PromethION (R.10.4.1) flow cells (Oxford Nanopore Technologies, Oxford, UK) for a run time of 72 hours, which achieved a comparable yield. On both instruments, for each sequencing run, positive controls (a mixture of Equid alphaherpesvirus 1 and Equine arteritis virus) and negative controls (NA isolated from Nuclease-Free Water (QIAGEN, Hilden, Germany)) were added.

### 2.7 Bioinformatics

All bioinformatic analyses were performed with a pipeline developed in-house, as previously published (Bosilj et al., 2024). First, adapter sequences were removed using BBDuk (v.39.01) (Bushnell, 2014) and host depletion was performed using bowtie2 (v2.50) (Langmead and Salzberg, 2012) by mapping trimmed reads to the human genome (GRCh38). Read classification was performed with the KrakenUniq (v1.0.2) (Breitwieser et al., 2018) tool. KronaTools (v.2.8.1) (Ondov et al., 2011) and Pavian (v1.0) (Breitwieser and Salzberg, 2020) were used to visualize KrakenUniq results. Because the pathogens in question were previously confirmed with molecular tests, during manual analysis of results we considered one read as detected and mNGS-positive (mREV(+)). If no reads were detected for the target pathogen, the results were considered as mNGS-negative (mREV(-)).

### 2.8 Automated score-based result ranking: ClinSeq score

From the KrakenUniq tool output file, the number of classified reads per taxon and *k*mer count, duplicity, and coverage were incorporated into a scoring system (ClinSeq score; CS), which allows ranking the results (Equation 1) and is calculated as follows:


(1) $$ClinSeq\;=\;\{log_{10}\left(R_{norm}^{w}\times k\right)\;|\;k=\frac{log_{10}\left(K+1\right)\;\times \;C}{D}\}$$

$R_{norm}^{w}$ represents the normalized read counts per 10 million total reads, where *w* is the weight factor, which can be adjusted (in this study *w* = 2 was used), *K* is the *k*mer count, *C* is the *k*mer coverage, and *D* is the duplicity of *k*mers. Furthermore, the presence of individual pathogens was assessed on an intra-run, cross samples basis. Microorganisms detected in more than 90% of the samples in the same run were flagged as background noise (likely contaminants) but not excluded from analysis. In addition, further data polishing was performed based on the reads present in the negative control. The top five ClinSeq score ranks were classified as likely or unlikely true positive. The ClinSeq score uses an adaptive threshold of 0.6× standard deviation above the mean. The adaptive threshold was determined experimentally by iterative testing by balancing CS(+)/CS(-) with manual analysis mREV(+)/mREV(-) detections.

### 2.9 Statistical analysis

Differences between groups were evaluated using the Wilcoxon rank sum (Mann–Whitney) test. The differences were considered significant at *p*< 0.05. Cohen’s kappa was employed to calculate the agreement between manually analyzed mNGS results and the ClinSeq score.

## 3 Results

### 3.1 Clinical sensitivity of developed mNGS workflow

**Figure 1:**
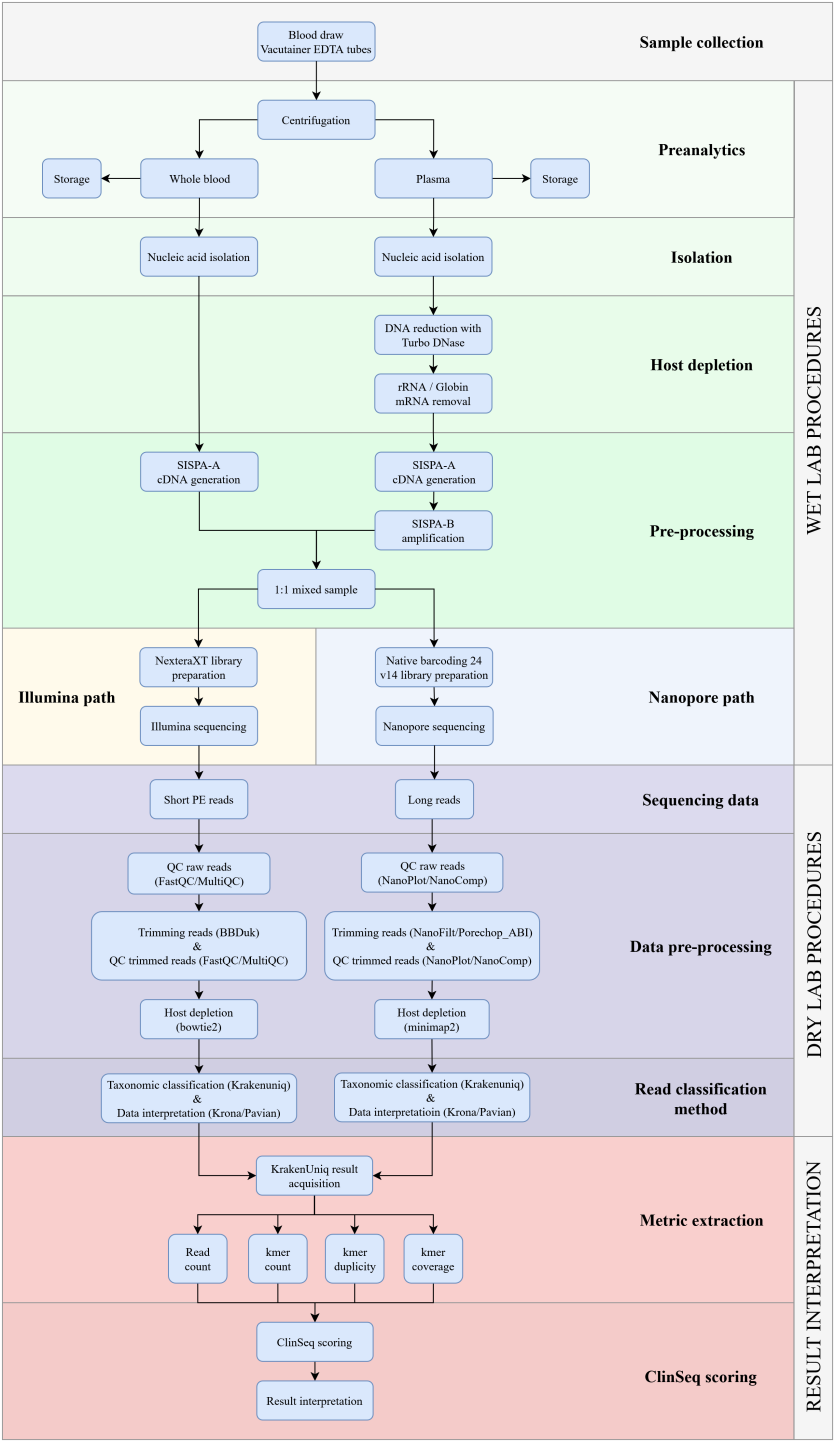
Detailed overview of the clinical metagenomics NGS workflow for identifying pathogens in EDTA blood from patients with undifferentiated fever. The workflow is divided into three sections: the laboratory protocol, the bioinformatics pipeline, and mNGS results analysis. The bioinformatics pipeline has been described in detail previously (Bosilj et al., 2024).

We developed a comprehensive mNGS workflow to detect a wide range of pathogens in patients with acute undifferentiated fever and no obvious localization of infection. First, to minimize the human background and increase the detection of RNA viruses, an EDTA blood sample was centrifuged to separate the plasma from the cells. After this, the plasma isolate was subjected to a series of treatment steps and amplified using the SISPA-B protocol. The whole blood isolate was left untreated and processed with the SISPA-A step. Finally, both fractions were mixed in equal volumes prior to NGS library construction. This strategy enhances the detection of DNA pathogens, particularly intracellular bacteria, and the treated plasma fraction guarantees amplification of RNA pathogens and minimization of the host background (**Figure 1**).

**Figure 2:**
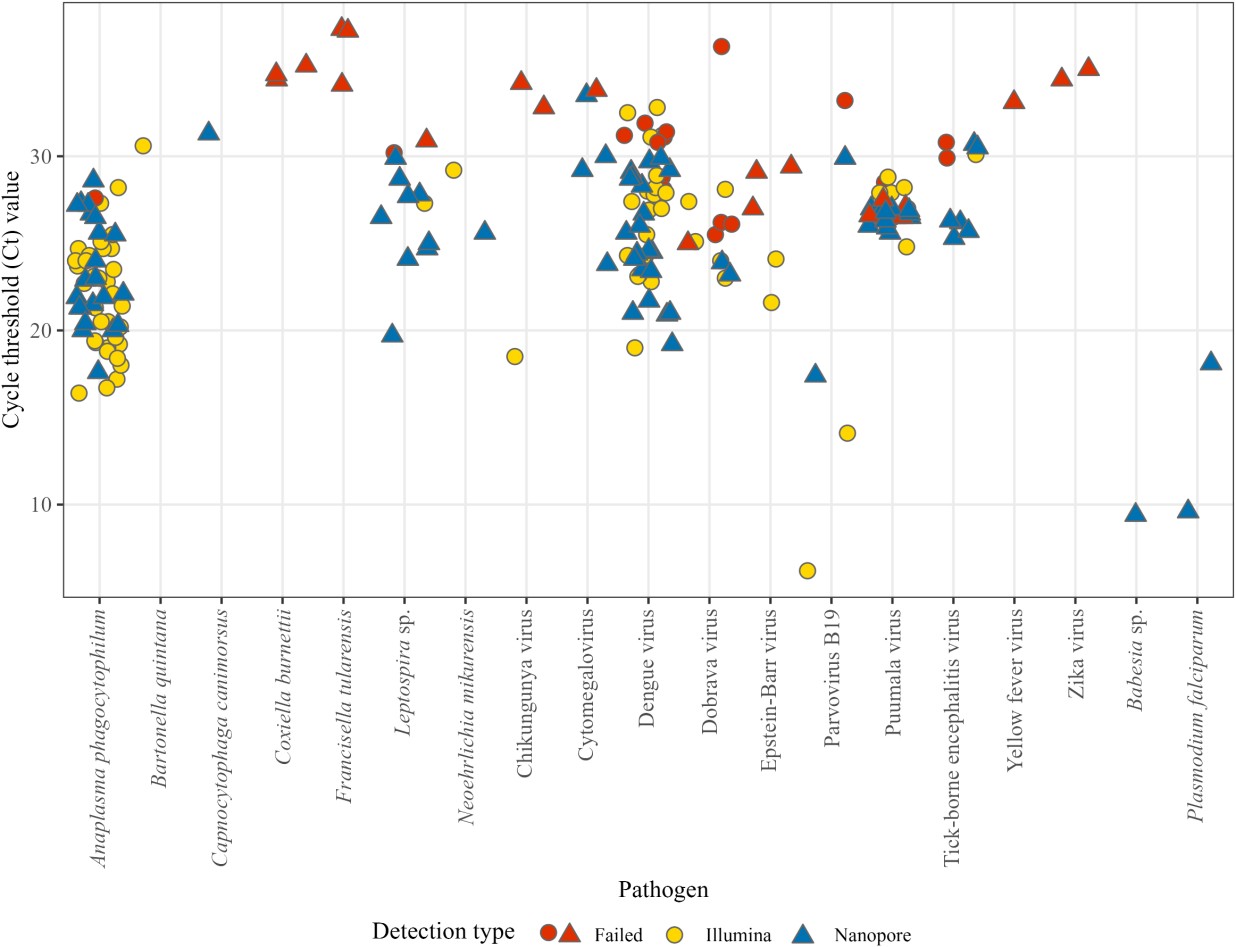
The performance of the developed mNGS workflow depending on the relative abundance of the pathogen, represented by Ct value from standardized *in vitro* diagnostic methods. Blue (n = 83) are mNGS positives with ONT, yellow (n = 76) are mNGS positives with Illumina and red (n = 41) represents failed mNGS detections.

We sequenced 200 samples from patients with acute undifferentiated fever with known etiology either with the Illumina or ONT platforms. In total, the mNGS workflow identified pathogens in 159 of the PCR positive samples, corresponding to a sensitivity of 79.5% (159/200). The mean Ct value of mNGS positive samples was 24.5 (range: 6.2 to 33.5). On the other hand, the mean Ct value of samples for which a pathogen was not detected by mNGS results was 30.6 (range: 25.0–37.3; **Figure 2**). Unlike other pathogens where Ct values were used as semi-quantitative indicators, EBV and CMV were quantified using IU/mL. For CMV, detected samples had viral loads ranging from 2.39 × 10³ to 2.16 × 10^6^ IU/mL (mean 5.59 × 10^5^ IU/mL), whereas samples that were not detected by mNGS had uniformly lower loads of 1.97 × 10³ IU/mL. For EBV, detected samples had viral loads ranging from 2.94 × 10^6^ to 1.81 × 10^7^ IU/mL (mean 1.05 × 10^7^ IU/mL), while mNGS negative samples ranged from 6.36 × 10^4^ to 3.45 × 10^5^ IU/mL (mean 1.62 × 10^5^ IU/mL) (**Table 1**).

**Table 1:** List of included EDTA blood samples collected from patients with undifferentiated fever and molecularly confirmed etiology of the disease.

Clinical diagnosis*	Age (mean)*	Sex (M%)*	Travel*	PCR confirmed pathogen	Total counts (Illu./ONT)	Mean Ct or IU/ml (min–max)
Bacterial infection	58.9	67.1	Y (0) N (79)	Bacterium	79 (39/40)	24.5 (16.4–37.3)
Anaplasmosis	58.4	59.6	Y (0) N (57)	*Anaplasma phagocytophilum*	57 (35/22)	22.5 (16.4–28.6)
Undifferentiated fever	82.0	0.0	Y (0) N (1)	*Bartonella quintana*	1 (1/0)	30.6
Undifferentiated fever	40.0	100.0	Y (0) N (1)	*Capnocytophaga canimorsus*	1 (0/1)	31.3
Q fever	65.3	100.0	Y (0) N (3)	*Coxiella burnettii*	3 (0/3)	34.8 (34.4–35.2)
Tularemia	63.7	100.0	Y (0) N (3)	*Francisella tularensis*	3 (0/3)	36.2 (34.1–37.3)
Leptospirosis	58.8	91.7	Y (0) N (12)	*Leptospira* sp.	12 (2/10)	26.9 (19.7–30.9)
Neoehrlichiosis	54.5	50.0	Y (0) N (2)	*Neoehrlichia mikurensis*	2 (1/1)	27.4 (25.6–29.2)
Parasitic infection	55.0	66.7	Y (2) N (1)	Parasite	3 (0/3)	12.4 (9.4–18.1)
Malaria	55.0	100.0	Y (2) N (0)	*Plasmodium falciparum*	2 (0/2)	13.9 (9.6–18.1)
Babesiosis	55.0	0.0	Y (0) N (1)	*Babesia* sp.	1 (0/1)	9.4 (9.4–9.4)
Viral infection	41.8	60.2	Y (53) N (65)	Virus	118 (56/62)	26.8 (6.2–36.6)
Chikungunya	29.7	66.7	Y (3) N (0)	Chikungunya virus	3 (1/2)	28.5 (18.5–34.2)
CMV fever	53.8	60.0	Y (0) N (5)	Cytomegalovirus	5 (0/5)	4.48 × 10^5^ IU/ml (1.97 × 10³–2.16 × 10^6^)
Dengue fever	36.8	47.9	Y (48) N (0)	Dengue virus	48 (27/21)	26.7 (19.0–32.8)
HFRS	38.9	75.0	Y (0) N (12)	Dobrava virus	12 (9/3)	26.2 (23.0–36.3)
Infectious mononucleosis	43.2	60.0	Y (0) N (5)	Epstein–Barr virus	5 (2/3)	4.30 × 10^6^ IU/ml (6.36 × 10^4^–1.81 × 10^7^)
Erythema infectiosum	25.6	60.0	Y (0) N (5)	Parvovirus B19	5 (3/2)	20.2 (6.2–33.2)
HFRS	52.8	75.0	Y (0) N (28)	Puumala virus	28 (11/17)	26.8 (24.8–28.8)
Tick-borne encephalitis	41.6	55.6	Y (0) N (9)	Tick-borne encephalitis virus	9 (3/6)	28.4 (25.3–30.8)
Yellow fever	64.0	100.0	Y (0) N (1)	Yellow fever virus	1 (0/1)	33.1
Zika	44.0	50.0	Y (2) N (0)	Zika virus	2 (0/2)	34.7 (34.4–35.0)
Total	63.0	48.8	Y (55) N (145)		200 (95/105)	25.7 (6.2–37.3)

*Individual patient data can be found in the **Supplementary Table S2**.

The table summarizes grouped patient demographics, travel history, PCR-confirmed pathogens, the number of samples sequenced using Illumina (Illu.) or Oxford Nanopore Technologies (ONT), and mean (min–max) cycle threshold (Ct) values or international units per milliliter (IU/ml).

For bacteria, the sensitivity was 88.6% (70/79). Successful pathogen detections by mNGS were as follows: *A. phagocytophilum* (56/57), *B. quintana*, *C. canimorsus*, *Leptospira* sp. (10/12), and *N. mikurensis*. Besides individual failed detections, we couldn’t detect cases with *C. burnettii* and *F. tularensis* infections. Both samples with *P. falciparum* and the one with *Babesia* sp. were successfully detected with mNGS. For DNA viruses, we observed sensitivity of 66.7% (10/15), while for RNA viruses, the sensitivity was 73.8% (76/103). Successful viral detections by mNGS were CMV (4/5), EBV (2/5), PB19 (4/5), CKIHV (1/3), DENV (40/48), DOBV (7/12), PUUV (21/28), and TBEV (7/9). Besides individual failed detections, unsuccessful detections were also in one YFV case and both ZIKV cases. Analyzing pathogen detections by individual sequencing methods, we observed the overall sensitivity of Illumina and ONT to be 80.0% (76/95) and 79.1% (83/105) respectively.

### 3.2 Automated ClinSeq score

With ClinSeq score we detected the pathogen in 126/200 samples, resulting in 63.0% sensitivity. The comparative evaluation of ClinSeq score and manual analysis showed an overall percent agreement of 83.5% (95% CI; 77.6%–88.4%), a positive percent agreement of 79.2% (95% CI; 72.1%–85.3%), a negative percent agreement of 100% (95% CI; 91.4%–100%), and a κ value of 0.61 (95% CI; 0.49–0.73), which reflects a substantial agreement between the two methods (**Table 2**).

**Table 2:** Comparison of mNGS manual analysis (mREV) and ClinSeq (CS) score, with calculated overall percent agreement (OPA), positive percent agreement (PPA), negative percent agreement (NPA), and Cohen’s kappa (κ).

		mREV			OPA	PPA	NPA	Cohen’s κ
		+	-	Total	(95% CI)	(95% CI)	(95% CI)	(95% CI)
CS	+	126	0	126	83.5%	79.2%	100%	0.61
	–	33	41	74				
					(77.6% – 88.4%)	(72.1% – 85.3%)	(91.4% – 100%)	(0.49 – 0.73)
	Total	159	41	200				

**Figure 3:**
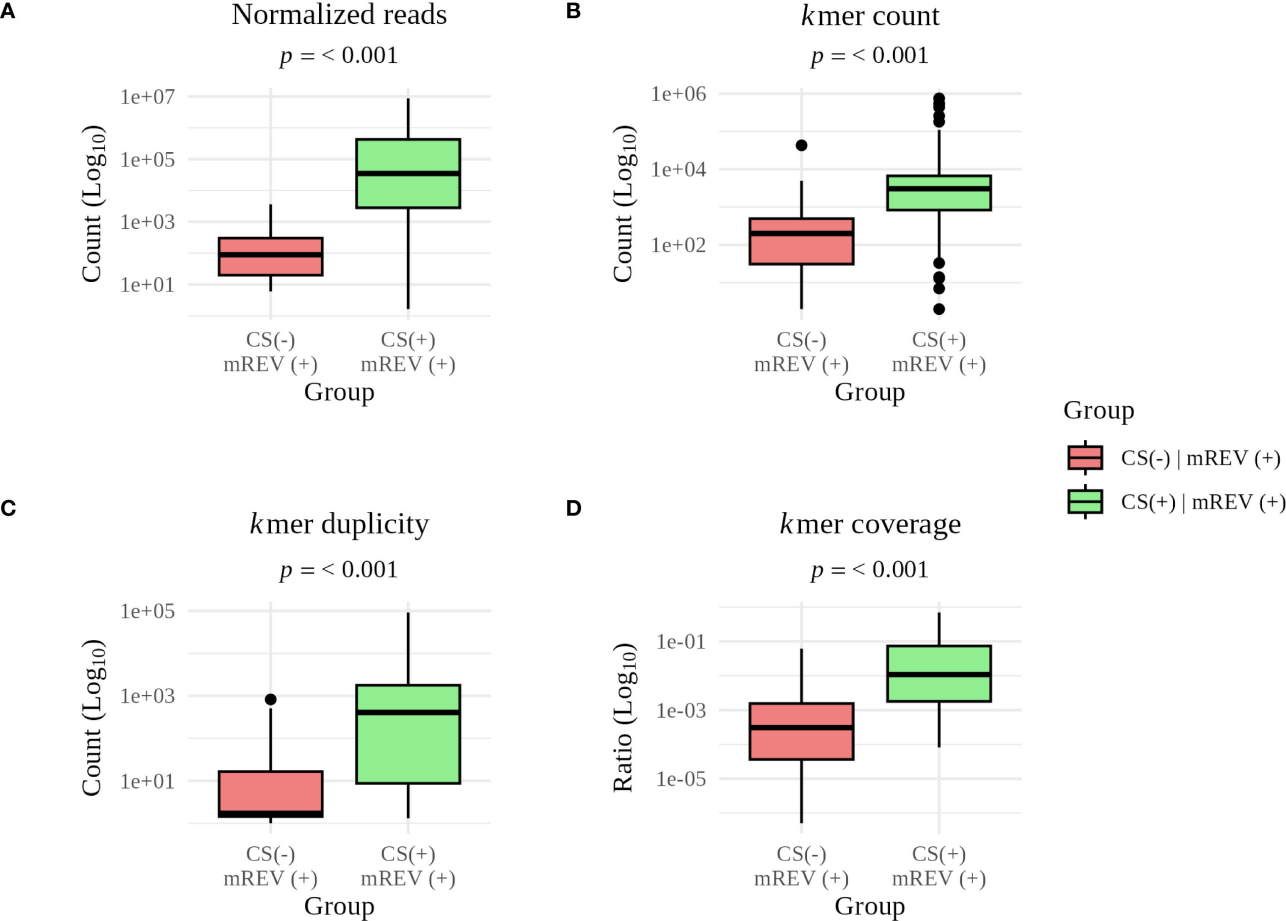
Comparison of individual metrics for concordant and discordant results between the ClinSeq score (CS) and manual analysis (mREV). Statistically significant differences are pathogen specific **(A)** read count (*p* = 1.9 × 10^-14^), **(B)** *k*mer count (*p* = 4.1 × 10^-9^), **(C)** *k*mer duplicity (*p* = 9.4 × 10^-10^), and **(D)** *k*mer coverage (*p* = 1.5 × 10^-11^). The long whiskers reflect the natural variability in read counts, kmer content, duplicity, and coverage within individual groups.

We investigated the reason for missed detections in the ClinSeq score (CS(-) | mREV(+)). When we compared the mean Ct between CS(-) and CS(+) samples, we observed a ΔCt of 2.8. This translated also to pathogen specific read counts, where we observed a statistically significant difference between CS(-) and CS(+) in normalized read counts, *k*mer count, duplicity and coverage (p< 0.001; **Figure 3**).

## 4 Discussion

The primary goal of this study was to develop a comprehensive mNGS workflow that maximizes pathogen detection while minimizing background noise, reducing costs, and ensuring a rapid turnaround time suitable for clinical applications. We developed an innovative approach to pre-processing EDTA blood samples, beginning with the separation of the sample into whole blood and plasma. These fractions undergo tailored pretreatment processes to optimize the enrichment of pathogen nucleic acids. Whole blood enables the detection of intracellular pathogens, including DNA viruses, intracellular bacteria and parasites. Conversely, the plasma fraction is processed separately to amplify pathogens with RNA genome. Because the fractions are mixed in equal volumes prior to library preparation, a reduction in hands-on time, turnaround time, cost, and complexity is achieved.

Overall, the developed mNGS workflow demonstrated a sensitivity of 79.5% (159/200) in comparison to conventional molecular diagnostic approach. This sensitivity is consistent with studies comparing blood pathogen detection with mNGS to droplet digital PCR (Hu et al., 2021) and to qPCR (Lu et al., 2022). The majority of standardized molecular methods for *in vitro* diagnostics are qualitative, so absolute quantification of the pathogen genome is not possible. However, the reported Ct values provide a relative estimation of the abundance of the pathogen (Kogoj et al., 2022). Bearing these limitations in mind, we compared the Ct values of mNGS-positive and -negative samples, observing that mNGS detection is less consistent when Ct > 30, a finding comparable to other studies (Gauthier et al., 2021, 2024; Koh et al., 2023; Pichler et al., 2023; Kandathil et al., 2024). Nevertheless, eleven samples with Ct > 30 in our dataset still yielded a reliable pathogen detection with mNGS. This indicates that other parameters also influence the success of detection. These include sample composition, nucleic acid integrity, pathogen type, amplification efficiency and biases introduced during procedures, such as the SISPA protocol (Bustin and Mueller, 2005; Rao et al., 2020). While the detection of potential pathogens can be improved with higher sequencing depth (Resman Rus et al., 2025), this however, comes at a higher cost, as a lower number of samples can be included per run.

Besides the challenge of sequencing the samples with a low pathogen load, they are equally fastidious for bioinformatics analysis, as their pathogen-to-background reads ratio is low. In the absence of standardized detection thresholds in such cases, interpretation becomes subjective and prone to bias. Although defining consistent thresholds is essential for objective data interpretation, the optimal cut-off point often varies depending on the pathogen and between sequencing runs, which presents a major analytical challenge. For this reason, we have developed an automated, score-based result ranking system called the ClinSeq score. This is a mathematical, data-driven algorithm that adapts to each sequencing run. Unlike fixed thresholds, the ClinSeq score sets an adaptive threshold defined as the mean read count plus 0.6× standard deviation for each classified taxon. Although manual mNGS analysis was ultimately superior to the ClinSeq score (79.5% versus 63.0%), the clinical microbiology interpretation was achieved in just under an hour after completing the bioinformatic workflow for 126 samples. Manual analysis, on the other hand, took between 30 minutes and 1 hour per sample, depending on the sample complexity and intra-run variability. Furthermore, as the ClinSeq score produced no false positive results, the manual analysis could focus solely on CS(-) samples, reducing labor and time. A similar mathematical algorithm was also developed by Guellil et al. and further modified by Borry (Guellil et al., 2022; Borry, 2022). However, when applied to our dataset both yielded lower sensitivity of 22.0% and 12.0%, respectively. While the PPA (79.2%) of our mNGS pipeline might not yet be considered as an excellent result and therefore suitable for replacing current microbiological methods, it is comparable to available data as summarized by Liu et al (Liu et al., 2025). Nevertheless, the benefits of the current mNGS pipeline are most prominent in the ability to detect pathogens which cannot be identified in blood cultures or need initial suspicion from the clinician.

## 5 Conclusion

In conclusion, the developed mNGS workflow demonstrates reliable and comprehensive detection of a wide range of pathogens, including uncultivable bacteria, viruses, and parasites, with promising sensitivity and efficiency. The innovative approach of combining plasma and whole blood samples prior to library preparation enhances detection capabilities while streamlining processing, reducing costs, and enabling flexible sequencing options. The implementation of a mathematical ranking method further improves interpretative efficiency. These findings provide a strong foundation for integrating mNGS into routine diagnostic settings. Future studies will focus on validating the workflow across additional sample types, as well as refining the analytic methods to optimize clinical utility.

## Data Availability

The datasets presented in this study can be found in online repositories. The names of the repository/repositories and accession number(s) can be found below: https://www.ncbi.nlm.nih.gov/bioproject, BioProject ID: PRJNA1275620.
