# Supplementary material for: Development and performance evaluation of a clinical metagenomics approach for identifying pathogens in the whole blood from patients with undifferentiated fever
Source: Front Cell Infect Microbiol. 2025 Sep 15;15:1667422. doi: 10.3389/fcimb.2025.1667422 (PMC12477237; doi:10.3389/fcimb.2025.1667422)
Supplement: Supplementary file 1 [file Table1.docx]

**Supplementary Table** **S1**: Supplementary data to article entitled: “Development and performance evaluation of a clinical metagenomics approach for identifying fastidious pathogens in the whole blood from patients with undifferentiated fever”. Table shows additional information regarding molecular tests used.

| Pathogen | Routine diagnostics method |
| --- | --- |
| Bacterium |  |
| *Anaplasma phagocytophilum* | (da Silva et al., 2016) |
| *Bartonella quintana* | (Ehrenborg et al., 2008) |
| *Coxiella burnettii* | (Panning et al., 2008b) |
| *Francisella tularensis* | (Versage et al., 2003) |
| *Leptospira* sp. | LightMix® Modular Leptospira, (Roche, Basel, Switzerland) |
| *Neoehrlichia mikurensis* | (Jahfari et al., 2012) |
| *Capnocytophaga canimorsus* | Real-time PCR assay targeting the 16S V3/V4 region (Molzym GmbH & Co. KG, Bremen, Germany) & Sanger Sequencing on 3500 Series Genetic Analyzer (Applied Biosystems, Foster City, CA, USA) |
| Parasite |  |
| *Plasmodium falciparum* | (Rougemont et al., 2004) |
| *Babesia* sp*.* | (Stańczak et al., 2015) |
| Virus |  |
| Chikungunya virus | (Panning et al., 2008a) |
| Cytomegalovirus | GeneProof® Cytomegalovirus (Cytomegalovirus) PCR Kit – (IVDR), (Geneproof, Brno, Czech Republic) |
| Dengue virus | (Huhtamo et al., 2010) |
| Dobrava virus | (Kramski et al., 2007) |
| Epstein–Barr virus | GeneProof® Epstein-Barr virus (Epstein-Barr virus) PCR Kit – (IVDR), (Geneproof, Brno, Czech Republic) |
| Parvovirus b19 | artus® Parvo B19 TM PCR kit, v.1, (QIAGEN, Hilden, Germany) |
| Puumala virus | (Kramski et al., 2007) |
| Tick-borne encephalitis virus | (Schwaiger and Cassinotti, 2003) |
| Yellow fever virus | (Drosten et al., 2002) |
| Zika virus | (Faye et al., 2013) |

Each assay was always supplemented with the appropriate quality control measures, namely positive control, negative control, and internal amplification control, to ensure the reliability and accuracy of the results.

**References to Supplementary Table S1**

da Silva, C. B., Pires, M. S., Vilela, J. A. R., Peckle, M., da Costa, R. L., Vitari, G. L. V., et al. (2016). A new quantitative PCR method for the detection of Anaplasma platys in dogs based on the citrate synthase gene. *Journal of Veterinary Diagnostic Investigation* 28, 529–535. doi: 10.1177/1040638716659101

Drosten, C., Göttig, S., Schilling, S., Asper, M., Panning, M., Schmitz, H., et al. (2002). Rapid detection and quantification of RNA of Ebola and Marburg viruses, Lassa virus, Crimean-Congo hemorrhagic fever virus, Rift Valley fever virus, dengue virus, and yellow fever virus by real-time reverse transcription-PCR. *J Clin Microbiol* 40, 2323–2330. doi: 10.1128/JCM.40.7.2323-2330.2002

Ehrenborg, C., Byström, R., Hjelm, E., Friman, G., and Holmberg, M. (2008). High Bartonella spp. seroprevalence in a Swedish homeless population but no evidence of trench fever. *Scand J Infect Dis* 40, 208–215. doi: 10.1080/00365540701632972

Faye, O., Faye, O., Diallo, D., Diallo, M., Weidmann, M., and Sall, A. A. (2013). Quantitative real-time PCR detection of Zika virus and evaluation with field-caught Mosquitoes. *Virol J* 10, 311. doi: 10.1186/1743-422X-10-311

Huhtamo, E., Hasu, E., Uzcátegui, N. Y., Erra, E., Nikkari, S., Kantele, A., et al. (2010). Early diagnosis of dengue in travelers: Comparison of a novel real-time RT-PCR, NS1 antigen detection and serology. *Journal of Clinical Virology* 47, 49–53. doi: 10.1016/J.JCV.2009.11.001

Jahfari, S., Fonville, M., Hengeveld, P., Reusken, C., Scholte, E. J., Takken, W., et al. (2012). Prevalence of Neoehrlichia mikurensis in ticks and rodents from North-west Europe. *Parasites & Vectors 2012 5:1* 5, 74. doi: 10.1186/1756-3305-5-74

Kramski, M., Meisel, H., Klempa, B., Krüger, D. H., Pauli, G., and Nitsche, A. (2007). Detection and Typing of Human Pathogenic Hantaviruses by Real-Time Reverse Transcription-PCR and Pyrosequencing. *Clin Chem* 53, 1899–1905. doi: 10.1373/CLINCHEM.2007.093245

Panning, M., Grywna, K., Van Esbroeck, M., Emmerich, P., and Drosten, C. (2008a). Chikungunya Fever in Travelers Returning to Europe from the Indian Ocean Region, 2006. *Emerg Infect Dis* 14, 416–422. doi: 10.3201/EID1403.070906

Panning, M., Kilwinski, J., Greiner-Fischer, S., Peters, M., Kramme, S., Frangoulidis, D., et al. (2008b). High throughput detection of Coxiella burnetii by real-time PCR with internal control system and automated DNA preparation. *BMC Microbiol* 8, 1–8. doi: 10.1186/1471-2180-8-77

Rougemont, M., Van Saanen, M., Sahli, R., Hinrikson, H. P., Bille, J., and Jaton, K. (2004). Detection of four Plasmodium species in blood from humans by 18S rRNA gene subunit-based and species-specific real-time PCR assays. *J Clin Microbiol* 42, 5636–5643. doi: 10.1128/JCM.42.12.5636-5643.2004

Schwaiger, M., and Cassinotti, P. (2003). Development of a quantitative real-time RT-PCR assay with internal control for the laboratory detection of tick borne encephalitis virus (TBEV) RNA. *Journal of Clinical Virology* 27, 136–145. doi: 10.1016/S1386-6532(02)00168-3

Stańczak, J., Cieniuch, S., Lass, A., Biernat, B., and Racewicz, M. (2015). Detection and quantification of Anaplasma phagocytophilum and Babesia spp. in Ixodes ricinus ticks from urban and rural environment, northern Poland, by real-time polymerase chain reaction. *Exp Appl Acarol* 66, 63–81. doi: 10.1007/S10493-015-9887-2

Versage, J. L., Severin, D. D. M., Chu, M. C., and Petersen, J. M. (2003). Development of a Multitarget Real-Time TaqMan PCR Assay for Enhanced Detection of Francisella tularensis in Complex Specimens. *J Clin Microbiol* 41, 5492–5499. doi: 10.1128/JCM.41.12.5492-5499.2003

**Supplementary Table** **S2**: Supplementary data to article entitled: “Development and performance evaluation of a clinical metagenomics approach for identifying fastidious pathogens in the whole blood from patients with undifferentiated fever”. Table shows additional detailed information on all samples included in this study.

| **ID** | **SRA_ID** | **SAMPLE_ID** | **Clinical diagnosis** | **Age** | **Sex** | **Travel history** | **PCR detected pathogen** | **Pathogen type** | **Ct** | **Sequencing method** | **ClinSeq** | **Manual** | **Total reads** | **Pathogen reads** | **Other reads** | **Ratio (host:pathogen)** | **Mean read length** | **Pathogen mean read length** |
| --- | --- | --- | --- | --- | --- | --- | --- | --- | --- | --- | --- | --- | --- | --- | --- | --- | --- | --- |
| **1** | S1 | 2024-NGS-1363 | Anaplasmosis | F | 62 | N | *Anaplasma phagocytophilum* | Bacteria | 16.40 | Illumina | CS(+) | mREV(+) | 6667134 | 11705 | 73575 | 513.12 | 149 | 128 |
| **2** | S2 | 2024-NGS-1364 | Anaplasmosis | M | 34 | N | *Anaplasma phagocytophilum* | Bacteria | 17.20 | Illumina | CS(+) | mREV(+) | 7022263 | 5654 | 93463 | 1115.47 | 149 | 128 |
| **3** | S3 | 2024-NGS-1365 | Anaplasmosis | M | 72 | N | *Anaplasma phagocytophilum* | Bacteria | 19.20 | Illumina | CS(+) | mREV(+) | 6263484 | 2127 | 84304 | 2630.68 | 149 | 129 |
| **4** | S4 | 2024-NGS-1368 | Anaplasmosis | M | 44 | N | *Anaplasma phagocytophilum* | Bacteria | 19.00 | Illumina | CS(+) | mREV(+) | 5708594 | 697 | 81626 | 7222.32 | 149 | 130 |
| **5** | S5 | 2024-NGS-1370 | Anaplasmosis | M | 61 | N | *Anaplasma phagocytophilum* | Bacteria | 22.80 | Illumina | CS(-) | mREV(+) | 7913029 | 70 | 106963 | 97574.83 | 149 | 131 |
| **6** | S6 | 2024-NGS-1910 | Anaplasmosis | M | 41 | N | *Anaplasma phagocytophilum* | Bacteria | 22.10 | Illumina | CS(+) | mREV(+) | 411621 | 31 | 7234 | 11231.97 | 149 | 123 |
| **7** | S7 | 2024-NGS-1911 | Anaplasmosis | F | 60 | N | *Anaplasma phagocytophilum* | Bacteria | 19.60 | Illumina | CS(+) | mREV(+) | 424758 | 80 | 11916 | 4211.67 | 149 | 104 |
| **8** | S8 | 2024-NGS-1914 | Anaplasmosis | F | 83 | N | *Anaplasma phagocytophilum* | Bacteria | 18.00 | Illumina | CS(+) | mREV(+) | 462847 | 522 | 15925 | 588.61 | 149 | 98 |
| **9** | S9 | 2024-NGS-1915 | Anaplasmosis | M | 22 | N | *Anaplasma phagocytophilum* | Bacteria | 19.30 | Illumina | CS(+) | mREV(+) | 582919 | 269 | 17464 | 1667.01 | 149 | 117 |
| **10** | S10 | 2024-NGS-1916 | Anaplasmosis | F | 74 | N | *Anaplasma phagocytophilum* | Bacteria | 24.00 | Illumina | CS(-) | mREV(+) | 439264 | 3 | 8483 | 143592.67 | 149 | 115 |
| **11** | S11 | 2024-NGS-1917 | Anaplasmosis | F | 47 | N | *Anaplasma phagocytophilum* | Bacteria | 19.40 | Illumina | CS(+) | mREV(+) | 351033 | 87 | 7003 | 3582.65 | 149 | 131 |
| **12** | S12 | 2024-NGS-1918 | Anaplasmosis | M | 44 | N | *Anaplasma phagocytophilum* | Bacteria | 18.80 | Illumina | CS(+) | mREV(+) | 431974 | 125 | 10909 | 2680.94 | 149 | 114 |
| **13** | S13 | 2024-NGS-1919 | Anaplasmosis | M | 69 | N | *Anaplasma phagocytophilum* | Bacteria | 20.50 | Illumina | CS(+) | mREV(+) | 407427 | 46 | 8882 | 8132.57 | 149 | 124 |
| **14** | S14 | 2024-NGS-1921 | Anaplasmosis | F | 58 | N | *Anaplasma phagocytophilum* | Bacteria | 23.70 | Illumina | CS(-) | mREV(+) | 460159 | 11 | 9544 | 37550.25 | 149 | 108 |
| **15** | S15 | 2024-NGS-1922 | Anaplasmosis | M | 80 | N | *Anaplasma phagocytophilum* | Bacteria | 16.70 | Illumina | CS(+) | mREV(+) | 3556611 | 614 | 103679 | 4633.81 | 149 | 125 |
| **16** | S16 | 2024-NGS-1923 | Anaplasmosis | M | 58 | N | *Anaplasma phagocytophilum* | Bacteria | 23.50 | Illumina | CS(-) | mREV(+) | 466940 | 6 | 13066 | 56733.25 | 149 | 122 |
| **17** | S17 | 2024-NGS-1924 | Anaplasmosis | M | 57 | N | *Anaplasma phagocytophilum* | Bacteria | 21.30 | Illumina | CS(-) | mREV(+) | 403559 | 27 | 7467 | 13202.07 | 149 | 137 |
| **18** | S18 | 2024-NGS-1925 | Anaplasmosis | F | 65 | N | *Anaplasma phagocytophilum* | Bacteria | 24.70 | Illumina | CS(-) | mREV(+) | 396517 | 1 | 9954 | 386562.00 | 149 | 149 |
| **19** | S19 | 2024-NGS-1926 | Anaplasmosis | F | 62 | N | *Anaplasma phagocytophilum* | Bacteria | 20.50 | Illumina | CS(+) | mREV(+) | 372219 | 25 | 7432 | 12158.57 | 149 | 126 |
| **20** | S20 | 2024-NGS-1927 | Anaplasmosis | F | 58 | N | *Anaplasma phagocytophilum* | Bacteria | 24.00 | Illumina | CS(-) | mREV(+) | 505548 | 1 | 9987 | 495560.00 | 149 | 140 |
| **21** | S21 | 2024-NGS-1928 | Anaplasmosis | M | 64 | N | *Anaplasma phagocytophilum* | Bacteria | 20.20 | Illumina | CS(-) | mREV(+) | 378243 | 20 | 6521 | 16160.83 | 149 | 120 |
| **22** | S22 | 2024-NGS-1929 | Anaplasmosis | F | 56 | N | *Anaplasma phagocytophilum* | Bacteria | 24.70 | Illumina | CS(-) | mREV(+) | 467123 | 3 | 12211 | 113727.00 | 149 | 125 |
| **23** | S23 | 2024-NGS-4399 | Anaplasmosis | F | 76 | N | *Anaplasma phagocytophilum* | Bacteria | 24.70 | Illumina | CS(+) | mREV(+) | 8421609 | 3003419 | 1766303 | 0.96 | 149 | 122 |
| **24** | S24 | 2024-NGS-4400 | Anaplasmosis | M | 66 | N | *Anaplasma phagocytophilum* | Bacteria | 24.30 | Illumina | CS(+) | mREV(+) | 9001622 | 1435377 | 5518849 | 1.16 | 149 | 124 |
| **25** | S25 | 2024-NGS-4401 | Anaplasmosis | M | 66 | N | *Anaplasma phagocytophilum* | Bacteria | 25.10 | Illumina | CS(+) | mREV(+) | 7618738 | 79249 | 6035407 | 16.52 | 149 | 121 |
| **26** | S26 | 2024-NGS-4402 | Anaplasmosis | F | 35 | N | *Anaplasma phagocytophilum* | Bacteria | 24.00 | Illumina | CS(+) | mREV(+) | 5452220 | 29485 | 3237027 | 66.54 | 149 | 127 |
| **27** | S27 | 2024-NGS-4403 | Anaplasmosis | M | 72 | N | *Anaplasma phagocytophilum* | Bacteria | 23.10 | Illumina | CS(+) | mREV(+) | 7367518 | 238507 | 5096597 | 7.32 | 149 | 122 |
| **28** | S28 | 2024-NGS-4404 | Anaplasmosis | M | 63 | N | *Anaplasma phagocytophilum* | Bacteria | 27.30 | Illumina | CS(-) | mREV(+) | 6863250 | 361 | 187500 | 17072.53 | 149 | 104 |
| **29** | S29 | 2024-NGS-4405 | Anaplasmosis | F | 68 | N | *Anaplasma phagocytophilum* | Bacteria | 22.70 | Illumina | CS(+) | mREV(+) | 7951466 | 47605 | 4435330 | 61.67 | 149 | 119 |
| **30** | S30 | 2024-NGS-4406 | Anaplasmosis | M | 70 | N | *Anaplasma phagocytophilum* | Bacteria | 25.50 | Illumina | CS(+) | mREV(+) | 5288393 | 68124 | 2802532 | 31.47 | 149 | 125 |
| **31** | S31 | 2024-NGS-4407 | Anaplasmosis | F | 47 | N | *Anaplasma phagocytophilum* | Bacteria | 28.20 | Illumina | CS(-) | mREV(+) | 6459855 | 2252 | 4668829 | 687.06 | 149 | 117 |
| **32** | S32 | 2024-NGS-4408 | Anaplasmosis | M | 69 | N | *Anaplasma phagocytophilum* | Bacteria | 21.40 | Illumina | CS(+) | mREV(+) | 5774763 | 137171 | 4976489 | 4.05 | 149 | 120 |
| **33** | S33 | 2024-NGS-4409 | Anaplasmosis | F | 60 | N | *Anaplasma phagocytophilum* | Bacteria | 23.00 | Illumina | CS(+) | mREV(+) | 5826526 | 9911 | 2066290 | 319.07 | 149 | 118 |
| **34** | S34 | 2024-NGS-4410 | Anaplasmosis | M | 60 | N | *Anaplasma phagocytophilum* | Bacteria | 18.40 | Illumina | CS(+) | mREV(+) | 6028958 | 635340 | 1656908 | 5.07 | 149 | 122 |
| **35** | S35 | 2024-NGS-4411 | Anaplasmosis | M | 43 | N | *Anaplasma phagocytophilum* | Bacteria | 27.60 | Illumina | CS(-) | mREV(-) | 5893801 | 0 | 2374161 |  | 149 |  |
| **36** | S36 | 2024-NGS-4442 | Undifferentiated fever | F | 82 | N | *Bartonella* *quintana* | Bacteria | 30.60 | Illumina | CS(+) | mREV(+) | 4505834 | 49 | 3481735 | 15059.28 | 149 | 105 |
| **37** | S38 | 2024-NGS-4379 | Leptospirosis | M | 75 | N | *Leptospira* sp. | Bacteria | 30.20 | Illumina | CS(-) | mREV(-) | 4556076 | 0 | 3831617 |  | 149 |  |
| **38** | S42 | 2024-NGS-4446 | Leptospirosis | M | 51 | N | *Leptospira* sp. | Bacteria | 27.30 | Illumina | CS(+) | mREV(+) | 4396353 | 2252 | 2333221 | 808.07 | 149 | 125 |
| **39** | S44 | 2024-NGS-4377 | Neoehrlichiosis | M | 53 | N | *Neoehrlichia* mikurensis | Bacteria | 29.20 | Illumina | CS(+) | mREV(+) | 3574868 | 989 | 320629 | 2957.40 | 149 | 126 |
| **40** | S47 | 2024-NGS-4439 | Chikungunya | M | 38 | Y, Thailand | Chikungunya virus | Virus | 18.50 | Illumina | CS(+) | mREV(+) | 5325994 | 6306 | 5239715 | 4.85 | 149 | 77 |
| **41** | S48 | 2024-NGS-1366 | Dengue fever | F | 32 | Y, Sri Lanka | Dengue virus | Virus | 22.80 | Illumina | CS(+) | mREV(+) | 5932095 | 689 | 89941 | 8169.84 | 149 | 136 |
| **42** | S49 | 2024-NGS-1367 | Dengue fever | M | 31 | Y, Egypt | Dengue virus | Virus | 23.20 | Illumina | CS(+) | mREV(+) | 7028628 | 286 | 139196 | 22440.15 | 149 | 135 |
| **43** | S50 | 2024-NGS-1369 | Dengue fever | F | 52 | Y, Maldives | Dengue virus | Virus | 19.00 | Illumina | CS(+) | mREV(+) | 7462771 | 4259 | 148520 | 1629.83 | 149 | 137 |
| **44** | S51 | 2024-NGS-1932 | Dengue fever | M | 23 | Y, Australia, Singapore | Dengue virus | Virus | 25.50 | Illumina | CS(+) | mREV(+) | 343929 | 4 | 7175 | 84187.50 | 149 | 146 |
| **45** | S52 | 2024-NGS-1936 | Dengue fever | F | 80 | Y, Thailand | Dengue virus | Virus | 26.90 | Illumina | CS(-) | mREV(+) | 326920 | 1 | 7363 | 319556.00 | 149 | 148 |
| **46** | S53 | 2024-NGS-1937 | Dengue fever | F | 34 | Y, Indonesia | Dengue virus | Virus | 28.20 | Illumina | CS(-) | mREV(-) | 396257 | 0 | 9004 |  | 149 |  |
| **47** | S54 | 2024-NGS-1939 | Dengue fever | F | 67 | Y, Egypt | Dengue virus | Virus | 28.80 | Illumina | CS(-) | mREV(-) | 450425 | 0 | 10647 |  | 149 |  |
| **48** | S55 | 2024-NGS-1940 | Dengue fever | F | 23 | Y, N/A* | Dengue virus | Virus | 23.10 | Illumina | CS(+) | mREV(+) | 345973 | 5 | 8959 | 67401.80 | 149 | 148 |
| **49** | S56 | 2024-NGS-1941 | Dengue fever | F | 40 | Y, Dominica, Martinique | Dengue virus | Virus | 28.10 | Illumina | CS(-) | mREV(-) | 437028 | 0 | 10073 |  | 149 |  |
| **50** | S57 | 2024-NGS-1942 | Dengue fever | M | 18 | Y, Maldives | Dengue virus | Virus | 27.00 | Illumina | CS(+) | mREV(+) | 411817 | 3 | 8830 | 134328.00 | 149 | 126 |
| **51** | S58 | 2024-NGS-4412 | Dengue fever | F | 72 | Y, Eritrea | Dengue virus | Virus | 31.20 | Illumina | CS(+) | mREV(+) | 8349175 | 177184 | 4246634 | 20.57 | 149 | 126 |
| **52** | S59 | 2024-NGS-4413 | Dengue fever | F | 29 | Y, SE Asia | Dengue virus | Virus | 28.00 | Illumina | CS(+) | mREV(+) | 5613623 | 2137618 | 3006859 | 0.15 | 149 | 130 |
| **53** | S60 | 2024-NGS-4414 | Dengue fever | M | 55 | Y, Thailand | Dengue virus | Virus | 32.50 | Illumina | CS(+) | mREV(+) | 6164402 | 308399 | 5528968 | 0.93 | 149 | 131 |
| **54** | S61 | 2024-NGS-4415 | Dengue fever | F | 49 | Y, Thailand | Dengue virus | Virus | 24.30 | Illumina | CS(+) | mREV(+) | 3051521 | 1705566 | 1244142 | 0.00 | 149 | 132 |
| **55** | S62 | 2024-NGS-4416 | Dengue fever | F | 18 | Y, India | Dengue virus | Virus | 27.40 | Illumina | CS(+) | mREV(+) | 6452497 | 2883057 | 3096619 | 0.10 | 149 | 131 |
| **56** | S63 | 2024-NGS-4417 | Dengue fever | M | 67 | Y, Myanmar | Dengue virus | Virus | 24.30 | Illumina | CS(+) | mREV(+) | 5993839 | 3895343 | 1406148 | 0.09 | 149 | 126 |
| **57** | S64 | 2024-NGS-4418 | Dengue fever | M | 28 | Y, Malaysia | Dengue virus | Virus | 27.80 | Illumina | CS(+) | mREV(+) | 6473089 | 2958132 | 2921325 | 0.08 | 149 | 124 |
| **58** | S65 | 2024-NGS-4419 | Dengue fever | M | 38 | Y, West Africa | Dengue virus | Virus | 32.80 | Illumina | CS(+) | mREV(+) | 6334685 | 693097 | 4766523 | 1.14 | 149 | 131 |
| **59** | S66 | 2024-NGS-4420 | Dengue fever | F | 28 | Y, Philippines | Dengue virus | Virus | 28.20 | Illumina | CS(+) | mREV(+) | 6929094 | 1920715 | 2539867 | 1.14 | 149 | 129 |
| **60** | S67 | 2024-NGS-4421 | Dengue fever | M | 18 | Y, Kenya | Dengue virus | Virus | 28.90 | Illumina | CS(-) | mREV(+) | 11995084 | 17 | 3750770 | 458016.44 | 149 | 125 |
| **61** | S68 | 2024-NGS-4422 | Dengue fever | M | 35 | Y, Vietnam | Dengue virus | Virus | 27.90 | Illumina | CS(+) | mREV(+) | 6051890 | 2347512 | 3179410 | 0.12 | 149 | 127 |
| **62** | S69 | 2024-NGS-4423 | Dengue fever | F | 33 | Y, SE Asia | Dengue virus | Virus | 31.90 | Illumina | CS(-) | mREV(-) | 5751097 | 0 | 3765865 |  | 149 |  |
| **63** | S70 | 2024-NGS-4424 | Dengue fever | M | 19 | Y, India | Dengue virus | Virus | 31.10 | Illumina | CS(+) | mREV(+) | 7263132 | 9399 | 5917010 | 123.74 | 149 | 128 |
| **64** | S71 | 2024-NGS-4425 | Dengue fever | M | 38 | Y, Puerto Rico | Dengue virus | Virus | 31.20 | Illumina | CS(-) | mREV(-) | 6738418 | 0 | 4808718 |  | 149 |  |
| **65** | S72 | 2024-NGS-4426 | Dengue fever | F | 24 | Y, Mexico | Dengue virus | Virus | 31.10 | Illumina | CS(-) | mREV(-) | 7413769 | 0 | 6489116 |  | 149 |  |
| **66** | S73 | 2024-NGS-4427 | Dengue fever | F | 30 | Y, Cambodia, Vietnam | Dengue virus | Virus | 31.40 | Illumina | CS(-) | mREV(-) | 6705685 | 0 | 6133475 |  | 149 |  |
| **67** | S74 | 2024-NGS-4428 | Dengue fever | M | 29 | Y, Mexico, Cuba | Dengue virus | Virus | 30.80 | Illumina | CS(-) | mREV(-) | 7594990 | 0 | 5815532 |  | 149 |  |
| **68** | S75 | 2024-NGS-1944 | HFRS | M | 30 | N | Dobrava virus | Virus | 25.50 | Illumina | CS(-) | mREV(-) | 355224 | 0 | 7263 |  | 149 |  |
| **69** | S76 | 2024-NGS-4381 | HFRS | M | 25 | N | Dobrava virus | Virus | 28.10 | Illumina | CS(+) | mREV(+) | 6529869 | 15268 | 3122326 | 200.68 | 149 | 123 |
| **70** | S77 | 2024-NGS-4382 | HFRS | F | 35 | N | Dobrava virus | Virus | 23.00 | Illumina | CS(+) | mREV(+) | 9780145 | 48291 | 5995649 | 72.75 | 149 | 130 |
| **71** | S78 | 2024-NGS-4383 | HFRS | M | 48 | N | Dobrava virus | Virus | 24.00 | Illumina | CS(+) | mREV(+) | 9429424 | 25614 | 4011249 | 194.26 | 149 | 124 |
| **72** | S79 | 2024-NGS-4384 | HFRS | M | 18 | N | Dobrava virus | Virus | 25.10 | Illumina | CS(+) | mREV(+) | 5740257 | 3820 | 2942193 | 657.68 | 149 | 123 |
| **73** | S80 | 2024-NGS-4385 | HFRS | M | 58 | N | Dobrava virus | Virus | 26.20 | Illumina | CS(-) | mREV(-) | 6299979 | 0 | 2906292 |  | 149 |  |
| **74** | S81 | 2024-NGS-4386 | HFRS | M | 62 | N | Dobrava virus | Virus | 26.10 | Illumina | CS(-) | mREV(-) | 4465533 | 0 | 1358832 |  | 149 |  |
| **75** | S82 | 2024-NGS-4387 | HFRS | F | 25 | N | Dobrava virus | Virus | 27.40 | Illumina | CS(+) | mREV(+) | 6042696 | 630 | 3376590 | 3738.28 | 149 | 121 |
| **76** | S83 | 2024-NGS-4388 | HFRS | F | 46 | N | Dobrava virus | Virus | 36.30 | Illumina | CS(-) | mREV(-) | 7179111 | 0 | 195062 |  | 149 |  |
| **77** | S88 | 2024-NGS-4433 | Undifferentiated fever without localization | F | 68 | N | Epstein-Barr virus | Virus | 21.60 | Illumina | CS(+) | mREV(+) | 6136361 | 267 | 1088124 | 13606.11 | 149 | 108 |
| **78** | S89 | 2024-NGS-4434 | Undifferentiated fever without localization | M | 69 | N | Epstein-Barr virus | Virus | 24.10 | Illumina | CS(+) | mREV(+) | 7693645 | 20 | 3055344 | 136419.62 | 149 | 96 |
| **79** | S90 | 2024-NGS-4378 | Erythema infectiosum | M | 18 | N | Parvovirus B19 | Virus | 6.20 | Illumina | CS(+) | mREV(+) | 6406376 | 21899 | 3412428 | 113.30 | 149 | 120 |
| **80** | S93 | 2024-NGS-4437 | Erythema infectiosum | M | 18 | N | Parvovirus B19 | Virus | 14.10 | Illumina | CS(+) | mREV(+) | 6055286 | 1764 | 4542660 | 740.48 | 149 | 121 |
| **81** | S94 | 2024-NGS-4438 | Erythema infectiosum | F | 18 | N | Parvovirus B19 | Virus | 33.20 | Illumina | CS(-) | mREV(-) | 6224953 | 0 | 920393 |  | 149 |  |
| **82** | S95 | 2024-NGS-1935 | HFRS | F | 31 | N | Puumala virus | Virus | 27.00 | Illumina | CS(-) | mREV(-) | 227612 | 0 | 4734 |  | 149 |  |
| **83** | S96 | 2024-NGS-4389 | HFRS | M | 28 | N | Puumala virus | Virus | 26.90 | Illumina | CS(+) | mREV(+) | 6840313 | 584 | 4092438 | 4578.79 | 149 | 117 |
| **84** | S97 | 2024-NGS-4390 | HFRS | M | 48 | N | Puumala virus | Virus | 28.50 | Illumina | CS(-) | mREV(-) | 5348247 | 0 | 3092049 |  | 149 |  |
| **85** | S98 | 2024-NGS-4391 | HFRS | F | 47 | N | Puumala virus | Virus | 27.90 | Illumina | CS(-) | mREV(+) | 3695439 | 89 | 1753946 | 19221.70 | 149 | 122 |
| **86** | S99 | 2024-NGS-4392 | HFRS | M | 60 | N | Puumala virus | Virus | 27.90 | Illumina | CS(-) | mREV(+) | 11337437 | 25 | 5868428 | 182299.30 | 149 | 129 |
| **87** | S100 | 2024-NGS-4393 | HFRS | M | 36 | N | Puumala virus | Virus | 24.80 | Illumina | CS(+) | mREV(+) | 6548250 | 4263 | 2067281 | 1001.90 | 149 | 136 |
| **88** | S101 | 2024-NGS-4394 | HFRS | M | 46 | N | Puumala virus | Virus | 26.50 | Illumina | CS(+) | mREV(+) | 6543345 | 831 | 2462153 | 4806.06 | 149 | 133 |
| **89** | S102 | 2024-NGS-4395 | HFRS | M | 58 | N | Puumala virus | Virus | 25.80 | Illumina | CS(+) | mREV(+) | 10499276 | 13631 | 6518706 | 279.88 | 149 | 133 |
| **90** | S103 | 2024-NGS-4396 | HFRS | M | 46 | N | Puumala virus | Virus | 28.80 | Illumina | CS(-) | mREV(+) | 7785492 | 153 | 1985828 | 36474.87 | 149 | 133 |
| **91** | S104 | 2024-NGS-4397 | HFRS | M | 57 | N | Puumala virus | Virus | 28.20 | Illumina | CS(+) | mREV(+) | 7774411 | 2795 | 5717143 | 698.99 | 149 | 128 |
| **92** | S105 | 2024-NGS-4398 | HFRS | M | 64 | N | Puumala virus | Virus | 26.60 | Illumina | CS(+) | mREV(+) | 6553040 | 420 | 1698907 | 11261.49 | 149 | 136 |
| **93** | S106 | 2024-NGS-4374 | Tick-borne encephalitis | M | 64 | N | Tick-borne encephalitis virus | Virus | 30.80 | Illumina | CS(-) | mREV(-) | 9737980 | 0 | 7492209 |  | 149 |  |
| **94** | S107 | 2024-NGS-4375 | Tick-borne encephalitis | M | 60 | N | Tick-borne encephalitis virus | Virus | 30.10 | Illumina | CS(+) | mREV(+) | 6240559 | 559 | 2543381 | 5731.06 | 149 | 116 |
| **95** | S108 | 2024-NGS-4376 | Tick-borne encephalitis | M | 18 | N | Tick-borne encephalitis virus | Virus | 29.90 | Illumina | CS(-) | mREV(-) | 6424281 | 0 | 557418 |  | 149 |  |
| **96** | S110 | 2024-NGS-3600 | Anaplasmosis | F | 47 | N | *Anaplasma phagocytophilum* | Bacteria | 21.30 | Nanopore | CS(+) | mREV(+) | 623348 | 30 | 554331 | 2299.57 | 612 | 787 |
| **97** | S111 | 2024-NGS-3601 | Anaplasmosis | M | 61 | N | *Anaplasma phagocytophilum* | Bacteria | 21.50 | Nanopore | CS(+) | mREV(+) | 2222413 | 23603 | 1582926 | 26.06 | 436 | 562 |
| **98** | S112 | 2024-NGS-3602 | Anaplasmosis | F | 50 | N | *Anaplasma phagocytophilum* | Bacteria | 21.90 | Nanopore | CS(+) | mREV(+) | 2342644 | 16338 | 1733661 | 36.23 | 433 | 548 |
| **99** | S113 | 2024-NGS-3603 | Anaplasmosis | F | 63 | N | *Anaplasma phagocytophilum* | Bacteria | 21.30 | Nanopore | CS(+) | mREV(+) | 2243877 | 18313 | 1964489 | 14.24 | 422 | 540 |
| **100** | S114 | 2024-NGS-3783 | Anaplasmosis | M | 28 | N | *Anaplasma phagocytophilum* | Bacteria | 25.60 | Nanopore | CS(-) | mREV(+) | 183769 | 1 | 166085 | 17683.00 | 436 | 172 |
| **101** | S115 | 2024-NGS-3784 | Anaplasmosis | M | 60 | N | *Anaplasma phagocytophilum* | Bacteria | 27.30 | Nanopore | CS(+) | mREV(+) | 793394 | 2805 | 709010 | 29.07 | 529 | 346 |
| **102** | S116 | 2024-NGS-3791 | Anaplasmosis | F | 55 | N | *Anaplasma phagocytophilum* | Bacteria | 20.00 | Nanopore | CS(+) | mREV(+) | 1202677 | 53508 | 394741 | 14.09 | 440 | 390 |
| **103** | S117 | 2024-NGS-3802 | Anaplasmosis | F | 51 | N | *Anaplasma phagocytophilum* | Bacteria | 26.70 | Nanopore | CS(+) | mREV(+) | 3267615 | 22019 | 2674569 | 25.93 | 531 | 341 |
| **104** | S118 | 2024-NGS-3810 | Anaplasmosis | M | 79 | N | *Anaplasma phagocytophilum* | Bacteria | 22.90 | Nanopore | CS(+) | mREV(+) | 951291 | 6297 | 778560 | 26.43 | 1069 | 434 |
| **105** | S119 | 2024-NGS-3818 | Anaplasmosis | M | 42 | N | *Anaplasma phagocytophilum* | Bacteria | 25.50 | Nanopore | CS(+) | mREV(+) | 2725000 | 65353 | 2174510 | 7.42 | 526 | 423 |
| **106** | S120 | 2024-NGS-3828 | Anaplasmosis | F | 74 | N | *Anaplasma phagocytophilum* | Bacteria | 24.00 | Nanopore | CS(+) | mREV(+) | 1124214 | 72 | 1003528 | 1675.19 | 659 | 749 |
| **107** | S121 | 2024-NGS-3834 | Anaplasmosis | M | 62 | N | *Anaplasma phagocytophilum* | Bacteria | 20.30 | Nanopore | CS(+) | mREV(+) | 1122366 | 185 | 1041157 | 433.27 | 628 | 482 |
| **108** | S122 | 2024-NGS-3835 | Anaplasmosis | M | 69 | N | *Anaplasma phagocytophilum* | Bacteria | 23.00 | Nanopore | CS(+) | mREV(+) | 1248830 | 2741 | 1007295 | 87.12 | 585 | 325 |
| **109** | S123 | 2024-NGS-3841 | Anaplasmosis | F | 52 | N | *Anaplasma phagocytophilum* | Bacteria | 27.20 | Nanopore | CS(-) | mREV(+) | 987787 | 1 | 936583 | 51203.00 | 589 | 238 |
| **110** | S124 | 2024-NGS-3842 | Anaplasmosis | M | 71 | N | *Anaplasma phagocytophilum* | Bacteria | 26.50 | Nanopore | CS(-) | mREV(+) | 1948447 | 3 | 1321317 | 209042.33 | 538 | 666 |
| **111** | S125 | 2024-NGS-3932 | Anaplasmosis | M | 68 | N | *Anaplasma phagocytophilum* | Bacteria | 27.20 | Nanopore | CS(+) | mREV(+) | 3245034 | 61982 | 1930635 | 20.18 | 482 | 518 |
| **112** | S126 | 2024-NGS-4189 | Anaplasmosis | M | 63 | N | *Anaplasma phagocytophilum* | Bacteria | 20.00 | Nanopore | CS(+) | mREV(+) | 693799 | 6252 | 588624 | 15.81 | 756 | 359 |
| **113** | S127 | 2024-NGS-4190 | Anaplasmosis | M | 36 | N | *Anaplasma phagocytophilum* | Bacteria | 28.60 | Nanopore | CS(-) | mREV(+) | 384182 | 139 | 287515 | 694.45 | 1206 | 508 |
| **114** | S128 | 2024-NGS-4191 | Anaplasmosis | M | 68 | N | *Anaplasma phagocytophilum* | Bacteria | 22.10 | Nanopore | CS(+) | mREV(+) | 1462190 | 57761 | 1097162 | 5.32 | 718 | 413 |
| **115** | S129 | 2024-NGS-4193 | Anaplasmosis | F | 52 | N | *Anaplasma phagocytophilum* | Bacteria | 21.90 | Nanopore | CS(+) | mREV(+) | 1464849 | 11543 | 1038281 | 35.95 | 643 | 403 |
| **116** | S130 | 2024-NGS-4194 | Anaplasmosis | M | 38 | N | *Anaplasma phagocytophilum* | Bacteria | 20.40 | Nanopore | CS(+) | mREV(+) | 819557 | 189893 | 368912 | 1.37 | 694 | 373 |
| **117** | S131 | 2024-NGS-4196 | Anaplasmosis | M | 72 | N | *Anaplasma phagocytophilum* | Bacteria | 17.60 | Nanopore | CS(+) | mREV(+) | 1189308 | 43132 | 809204 | 7.80 | 623 | 370 |
| **118** | S133 | 2024-NGS-3930 | Undifferentiated fever | M | 40 | N | *Capnocytophaga* canimorsus | Bacteria | 31.30 | Nanopore | CS(+) | mREV(+) | 6115833 | 1 | 2891769 | 3224063.00 | 469 | 2984 |
| **119** | S134 | 2024-NGS-3919 | Q fever | M | 57 | N | *Coxiella* *burnettii* | Bacteria | 34.40 | Nanopore | CS(-) | mREV(-) | 1282146 | 0 | 1032905 |  | 581 |  |
| **120** | S135 | 2024-NGS-3921 | Q fever | M | 53 | N | *Coxiella* *burnettii* | Bacteria | 34.70 | Nanopore | CS(-) | mREV(-) | 5183538 | 0 | 3706740 |  | 446 |  |
| **121** | S136 | 2024-NGS-3926 | Q fever | M | 86 | N | *Coxiella* *burnettii* | Bacteria | 35.20 | Nanopore | CS(-) | mREV(-) | 479197 | 0 | 335240 |  | 840 |  |
| **122** | S138 | 2024-NGS-3946 | Leptospirosis | M | 56 | N | *Leptospira* sp. | Bacteria | 27.80 | Nanopore | CS(-) | mREV(+) | 1426050 | 707 | 1025711 | 565.25 | 723 | 340 |
| **123** | S142 | 2024-NGS-3950 | Leptospirosis | M | 56 | N | *Leptospira* sp. | Bacteria | 27.70 | Nanopore | CS(+) | mREV(+) | 137930 | 25 | 75976 | 2477.16 | 643 | 374 |
| **124** | S143 | 2024-NGS-3951 | Leptospirosis | M | 54 | N | *Leptospira* sp. | Bacteria | 26.50 | Nanopore | CS(+) | mREV(+) | 16801 | 2 | 9936 | 3431.50 | 801 | 560 |
| **125** | S144 | 2024-NGS-3952 | Leptospirosis | M | 39 | N | *Leptospira* sp. | Bacteria | 30.90 | Nanopore | CS(-) | mREV(-) | 3327751 | 0 | 2582025 |  | 660 |  |
| **126** | S146 | 2024-NGS-3954 | Leptospirosis | F | 61 | N | *Leptospira* sp. | Bacteria | 24.10 | Nanopore | CS(+) | mREV(+) | 4705499 | 5451 | 2485163 | 405.06 | 487 | 510 |
| **127** | S147 | 2024-NGS-3955 | Leptospirosis | M | 65 | N | *Leptospira* sp. | Bacteria | 24.70 | Nanopore | CS(+) | mREV(+) | 533221 | 167 | 296292 | 1417.74 | 809 | 501 |
| **128** | S149 | 2024-NGS-4198 | Leptospirosis | M | 47 | N | *Leptospira* sp. | Bacteria | 19.70 | Nanopore | CS(+) | mREV(+) | 2361873 | 14641 | 1829801 | 35.21 | 526 | 476 |
| **129** | S150 | 2024-NGS-4199 | Leptospirosis | M | 87 | N | *Leptospira* sp. | Bacteria | 28.70 | Nanopore | CS(-) | mREV(+) | 766817 | 5 | 635139 | 26334.60 | 762 | 186 |
| **130** | S151 | 2024-NGS-4200 | Leptospirosis | M | 63 | N | *Leptospira* sp. | Bacteria | 29.90 | Nanopore | CS(-) | mREV(+) | 9438771 | 1198 | 7756390 | 1402.15 | 474 | 329 |
| **131** | S152 | 2024-NGS-4201 | Leptospirosis | M | 51 | N | *Leptospira* sp. | Bacteria | 25.00 | Nanopore | CS(+) | mREV(+) | 1704761 | 62 | 947074 | 12219.76 | 587 | 385 |
| **132** | S153 | 2024-NGS-3935 | Neoehrlichiosis | F | 56 | N | *Neoehrlichia* *mikurensis* | Bacteria | 25.60 | Nanopore | CS(+) | mREV(+) | 9159605 | 3952462 | 2654612 | 0.64 | 503 | 528 |
| **133** | S160 | 2024-NGS-3945 | Tularemia | M | 68 | N | *Francisella tularensis* | Bacteria | 37.30 | Nanopore | CS(-) | mREV(-) | 6140551 | 0 | 3312671 |  | 486 |  |
| **134** | S161 | 2024-NGS-3957 | Tularemia | M | 77 | N | *Francisella tularensis* | Bacteria | 37.20 | Nanopore | CS(-) | mREV(-) | 1699425 | 0 | 1494670 |  | 691 |  |
| **135** | S162 | 2024-NGS-3958 | Tularemia | M | 46 | N | *Francisella tularensis* | Bacteria | 34.10 | Nanopore | CS(-) | mREV(-) | 891021 | 0 | 481470 |  | 715 |  |
| **136** | S163 | 2024-NGS-3944 | Babesiosis | F | 55 | N | *Babesia* sp. | Parasite | 9.40 | Nanopore | CS(+) | mREV(+) | 6535403 | 1048828 | 1569196 | 3.72 | 443 | 313 |
| **137** | S164 | 2024-NGS-3408 | Malaria | M | 58 | Y, Uganda | *Plasmodium falciparum* | Parasite | 18.10 | Nanopore | CS(+) | mREV(+) | 1969468 | 362541 | 658276 | 2.14 | 546 | 563 |
| **138** | S165 | 2024-NGS-3537 | Malaria | M | 52 | Y, Ivory coast | *Plasmodium falciparum* | Parasite | 9.60 | Nanopore | CS(+) | mREV(+) | 1102036 | 411434 | 118580 | 1.37 | 459 | 469 |
| **139** | S167 | 2024-NGS-3923 | Chikungunya | M | 29 | N | Chikungunya virus | Virus | 32.80 | Nanopore | CS(-) | mREV(-) | 522598 | 0 | 372841 |  | 833 |  |
| **140** | S168 | 2024-NGS-3924 | Chikungunya | F | 22 | N | Chikungunya virus | Virus | 34.20 | Nanopore | CS(-) | mREV(-) | 806016 | 0 | 544297 |  | 702 |  |
| **141** | S169 | 2024-NGS-3608 | Dengue fever | M | 29 | Y, Cuba | Dengue virus | Virus | 23.50 | Nanopore | CS(+) | mREV(+) | 3033233 | 1061889 | 1737442 | 0.22 | 455 | 523 |
| **142** | S170 | 2024-NGS-3609 | Dengue fever | F | 27 | Y, Malaysia | Dengue virus | Virus | 24.50 | Nanopore | CS(+) | mREV(+) | 4556986 | 3263272 | 1229860 | 0.02 | 509 | 542 |
| **143** | S171 | 2024-NGS-3610 | Dengue fever | F | 18 | Y, SE Asia | Dengue virus | Virus | 24.60 | Nanopore | CS(+) | mREV(+) | 3258467 | 563951 | 2619738 | 0.13 | 429 | 581 |
| **144** | S172 | 2024-NGS-3611 | Dengue fever | M | 48 | Y, Panama | Dengue virus | Virus | 24.40 | Nanopore | CS(+) | mREV(+) | 3591649 | 2776583 | 691023 | 0.04 | 512 | 532 |
| **145** | S173 | 2024-NGS-3782 | Dengue fever | M | 40 | Y, SE Asia | Dengue virus | Virus | 26.70 | Nanopore | CS(+) | mREV(+) | 689583 | 43307 | 587163 | 1.36 | 559 | 518 |
| **146** | S174 | 2024-NGS-3789 | Dengue fever | F | 38 | Y, Maldives | Dengue virus | Virus | 19.20 | Nanopore | CS(+) | mREV(+) | 1058179 | 5323 | 510326 | 101.92 | 466 | 340 |
| **147** | S175 | 2024-NGS-3790 | Dengue fever | F | 37 | Y, N/A* | Dengue virus | Virus | 25.60 | Nanopore | CS(+) | mREV(+) | 595942 | 47244 | 505788 | 0.91 | 553 | 475 |
| **148** | S176 | 2024-NGS-3797 | Dengue fever | M | 52 | Y, Maldives | Dengue virus | Virus | 28.30 | Nanopore | CS(+) | mREV(+) | 87776 | 140 | 82516 | 36.57 | 581 | 455 |
| **149** | S177 | 2024-NGS-3801 | Dengue fever | F | 31 | Y, Thailand | Dengue virus | Virus | 29.20 | Nanopore | CS(+) | mREV(+) | 1707604 | 5851 | 1584754 | 20.00 | 539 | 1102 |
| **150** | S178 | 2024-NGS-3808 | Dengue fever | M | 42 | Y, Thailand | Dengue virus | Virus | 21.00 | Nanopore | CS(+) | mREV(+) | 7694444 | 6610410 | 1037120 | 0.01 | 498 | 502 |
| **151** | S179 | 2024-NGS-3809 | Dengue fever | M | 26 | Y, Mexico | Dengue virus | Virus | 26.00 | Nanopore | CS(+) | mREV(+) | 3219408 | 48469 | 2911554 | 5.35 | 504 | 510 |
| **152** | S180 | 2024-NGS-3816 | Dengue fever | M | 18 | Y, Maldives | Dengue virus | Virus | 29.10 | Nanopore | CS(-) | mREV(+) | 2299935 | 211 | 2083704 | 1023.79 | 497 | 371 |
| **153** | S181 | 2024-NGS-3817 | Dengue fever | M | 68 | Y, Maldives | Dengue virus | Virus | 29.70 | Nanopore | CS(+) | mREV(+) | 1872430 | 6725 | 1756693 | 16.21 | 540 | 356 |
| **154** | S182 | 2024-NGS-3826 | Dengue fever | F | 25 | Y, Thailand | Dengue virus | Virus | 28.80 | Nanopore | CS(+) | mREV(+) | 2501452 | 108885 | 1872159 | 4.78 | 484 | 549 |
| **155** | S183 | 2024-NGS-3827 | Dengue fever | F | 46 | Y, Thailand | Dengue virus | Virus | 29.90 | Nanopore | CS(-) | mREV(+) | 1218445 | 2 | 1084176 | 67133.50 | 599 | 474 |
| **156** | S184 | 2024-NGS-3833 | Dengue fever | M | 47 | Y, Cuba | Dengue virus | Virus | 28.70 | Nanopore | CS(+) | mREV(+) | 1458084 | 22013 | 1330398 | 4.80 | 564 | 387 |
| **157** | S185 | 2024-NGS-3840 | Dengue fever | M | 28 | Y, Maldives | Dengue virus | Virus | 20.90 | Nanopore | CS(+) | mREV(+) | 1563269 | 367227 | 1008486 | 0.51 | 605 | 476 |
| **158** | S186 | 2024-NGS-4187 | Dengue fever | F | 50 | Y, Seychelles | Dengue virus | Virus | 23.40 | Nanopore | CS(+) | mREV(+) | 1792925 | 759182 | 998195 | 0.05 | 651 | 530 |
| **159** | S187 | 2024-NGS-4188 | Dengue fever | F | 56 | Y, Maldives | Dengue virus | Virus | 21.00 | Nanopore | CS(+) | mREV(+) | 13178129 | 11441468 | 1582862 | 0.01 | 569 | 547 |
| **160** | S188 | 2024-NGS-4192 | Dengue fever | M | 24 | Y, El Salvador | Dengue virus | Virus | 21.70 | Nanopore | CS(+) | mREV(+) | 1139454 | 457101 | 632211 | 0.11 | 773 | 512 |
| **161** | S189 | 2024-NGS-4195 | Dengue fever | F | 28 | Y, India | Dengue virus | Virus | 24.10 | Nanopore | CS(+) | mREV(+) | 2119088 | 50981 | 1961938 | 2.08 | 664 | 492 |
| **162** | S190 | 2024-NGS-4202 | HFRS | M | 38 | N | Dobrava virus | Virus | 23.90 | Nanopore | CS(-) | mREV(+) | 321410 | 1 | 246952 | 74457.00 | 1263 | 1067 |
| **163** | S191 | 2024-NGS-4203 | HFRS | M | 47 | N | Dobrava virus | Virus | 25.00 | Nanopore | CS(-) | mREV(-) | 1218211 | 0 | 1093296 |  | 624 |  |
| **164** | S192 | 2024-NGS-4204 | HFRS | M | 44 | N | Dobrava virus | Virus | 23.20 | Nanopore | CS(+) | mREV(+) | 1558836 | 625 | 1457408 | 161.28 | 640 | 321 |
| **165** | S193 | 2024-NGS-3596 | CMV hepatitis | M | 62 | N | Cytomegalovirus | Virus | 30.00 | Nanopore | CS(-) | mREV(+) | 3033865 | 64 | 2727494 | 4786.05 | 395 | 496 |
| **166** | S194 | 2024-NGS-3597 | CMV fever | F | 32 | N | Cytomegalovirus | Virus | 29.20 | Nanopore | CS(+) | mREV(+) | 3146323 | 130 | 2647752 | 3834.16 | 399 | 896 |
| **167** | S195 | 2024-NGS-3598 | CMV fever | M | 77 | N | Cytomegalovirus | Virus | 33.50 | Nanopore | CS(-) | mREV(+) | 1675479 | 1 | 1401207 | 274271.00 | 449 | 668 |
| **168** | S196 | 2024-NGS-3599 | CMV fever | F | 49 | N | Cytomegalovirus | Virus | 23.80 | Nanopore | CS(+) | mREV(+) | 2775409 | 2971 | 1942262 | 279.14 | 427 | 527 |
| **169** | S198 | 2024-NGS-3788 | Undifferentiated fever without localization | M | 49 | N | Cytomegalovirus | Virus | 33.80 | Nanopore | CS(-) | mREV(-) | 1129357 | 0 | 941973 |  | 443 |  |
| **170** | S208 | 2024-NGS-3787 | Encephalitis, mielitis | F | 24 | N | Epstein-Barr virus | Virus | 27.00 | Nanopore | CS(-) | mREV(-) | 490048 | 0 | 417098 |  | 528 |  |
| **171** | S216 | 2024-NGS-3830 | Infectious mononucleosis | M | 28 | N | Epstein-Barr virus | Virus | 29.40 | Nanopore | CS(-) | mREV(-) | 1729851 | 0 | 1213282 |  | 469 |  |
| **172** | S217 | 2024-NGS-3837 | Infectious mononucleosis | M | 27 | N | Epstein-Barr virus | Virus | 29.10 | Nanopore | CS(-) | mREV(-) | 2819898 | 0 | 2044011 |  | 428 |  |
| **173** | S219 | 2024-NGS-3825 | Erythema infectiosum | F | 64 | N | Parvovirus B19 | Virus | 17.40 | Nanopore | CS(+) | mREV(+) | 3837256 | 18226 | 1298185 | 138.29 | 441 | 374 |
| **174** | S220 | 2024-NGS-3832 | Erythema infectiosum | M | 27 | N | Parvovirus B19 | Virus | 29.90 | Nanopore | CS(-) | mREV(+) | 3228308 | 2 | 921018 | 1153644.00 | 474 | 454 |
| **175** | S222 | 2024-NGS-3604 | HFRS | F | 83 | N | Puumala virus | Virus | 26.00 | Nanopore | CS(+) | mREV(+) | 2561028 | 20770 | 1457684 | 52.12 | 427 | 633 |
| **176** | S223 | 2024-NGS-3605 | HFRS | M | 85 | N | Puumala virus | Virus | 26.50 | Nanopore | CS(+) | mREV(+) | 2689782 | 2319 | 1970015 | 309.38 | 400 | 552 |
| **177** | S224 | 2024-NGS-3606 | HFRS | M | 71 | N | Puumala virus | Virus | 25.60 | Nanopore | CS(+) | mREV(+) | 2485001 | 21029 | 1668292 | 37.84 | 414 | 585 |
| **178** | S225 | 2024-NGS-3607 | HFRS | M | 83 | N | Puumala virus | Virus | 26.50 | Nanopore | CS(+) | mREV(+) | 1947590 | 2148 | 1321788 | 290.34 | 415 | 570 |
| **179** | S226 | 2024-NGS-3785 | HFRS | M | 64 | N | Puumala virus | Virus | 26.50 | Nanopore | CS(-) | mREV(-) | 686465 | 0 | 511571 |  | 553 |  |
| **180** | S227 | 2024-NGS-3792 | HFRS | M | 30 | N | Puumala virus | Virus | 27.10 | Nanopore | CS(-) | mREV(-) | 846146 | 0 | 502344 |  | 467 |  |
| **181** | S228 | 2024-NGS-3793 | HFRS | M | 61 | N | Puumala virus | Virus | 26.90 | Nanopore | CS(-) | mREV(-) | 644558 | 0 | 350022 |  | 502 |  |
| **182** | S229 | 2024-NGS-3803 | HFRS | M | 54 | N | Puumala virus | Virus | 27.00 | Nanopore | CS(-) | mREV(+) | 2230533 | 763 | 1503131 | 952.34 | 497 | 403 |
| **183** | S230 | 2024-NGS-3804 | HFRS | F | 61 | N | Puumala virus | Virus | 26.90 | Nanopore | CS(-) | mREV(+) | 1803085 | 631 | 1535341 | 423.32 | 566 | 332 |
| **184** | S231 | 2024-NGS-3811 | HFRS | M | 27 | N | Puumala virus | Virus | 26.70 | Nanopore | CS(+) | mREV(+) | 2282450 | 829 | 2065230 | 261.03 | 553 | 352 |
| **185** | S232 | 2024-NGS-3812 | HFRS | F | 48 | N | Puumala virus | Virus | 27.00 | Nanopore | CS(+) | mREV(+) | 3174742 | 4119 | 1760429 | 342.36 | 467 | 623 |
| **186** | S233 | 2024-NGS-3819 | HFRS | M | 63 | N | Puumala virus | Virus | 25.90 | Nanopore | CS(+) | mREV(+) | 3410638 | 1748 | 2813280 | 340.54 | 466 | 363 |
| **187** | S234 | 2024-NGS-3829 | HFRS | F | 29 | N | Puumala virus | Virus | 26.60 | Nanopore | CS(-) | mREV(-) | 930528 | 0 | 858989 |  | 715 |  |
| **188** | S235 | 2024-NGS-3836 | HFRS | F | 38 | N | Puumala virus | Virus | 26.90 | Nanopore | CS(+) | mREV(+) | 2413341 | 2008 | 1353127 | 527.00 | 494 | 314 |
| **189** | S236 | 2024-NGS-3843 | HFRS | M | 64 | N | Puumala virus | Virus | 26.30 | Nanopore | CS(-) | mREV(+) | 430415 | 13 | 328477 | 7840.38 | 619 | 198 |
| **190** | S237 | 2024-NGS-4205 | HFRS | M | 53 | N | Puumala virus | Virus | 27.50 | Nanopore | CS(-) | mREV(-) | 482125 | 0 | 467201 |  | 830 |  |
| **191** | S238 | 2024-NGS-4206 | HFRS | M | 42 | N | Puumala virus | Virus | 26.80 | Nanopore | CS(-) | mREV(+) | 3793236 | 48 | 3110017 | 14232.73 | 533 | 256 |
| **192** | S239 | 2024-NGS-3612 | Tick-borne encephalitis | F | 52 | N | Tick-borne encephalitis virus | Virus | 26.20 | Nanopore | CS(+) | mREV(+) | 2794141 | 88294 | 2208773 | 5.63 | 417 | 585 |
| **193** | S240 | 2024-NGS-3613 | Tick-borne encephalitis | F | 55 | N | Tick-borne encephalitis virus | Virus | 25.30 | Nanopore | CS(+) | mREV(+) | 2302372 | 217434 | 1567618 | 2.38 | 440 | 567 |
| **194** | S241 | 2024-NGS-3614 | Tick-borne encephalitis | F | 21 | N | Tick-borne encephalitis virus | Virus | 26.30 | Nanopore | CS(+) | mREV(+) | 1970061 | 250816 | 1428172 | 1.16 | 465 | 609 |
| **195** | S242 | 2024-NGS-3615 | Tick-borne encephalitis | F | 57 | N | Tick-borne encephalitis virus | Virus | 25.70 | Nanopore | CS(+) | mREV(+) | 1580435 | 140220 | 682940 | 5.40 | 480 | 626 |
| **196** | S243 | 2024-NGS-3959 | Tick-borne encephalitis | M | 26 | N | Tick-borne encephalitis virus | Virus | 30.70 | Nanopore | CS(+) | mREV(+) | 1216532 | 363 | 668038 | 1510.00 | 691 | 589 |
| **197** | S244 | 2024-NGS-3960 | Tick-borne encephalitis | M | 28 | N | Tick-borne encephalitis virus | Virus | 30.50 | Nanopore | CS(+) | mREV(+) | 64618 | 848 | 50038 | 16.19 | 1340 | 681 |
| **198** | S245 | 2024-NGS-3920 | Yellow fever, after vaccination | M | 64 | N | Yellow fever virus | Virus | 33.10 | Nanopore | CS(-) | mREV(-) | 2562143 | 0 | 2081751 |  | 455 |  |
| **199** | S246 | 2024-NGS-3925 | Zika | M | 40 | Y, India | Zika virus | Virus | 35.00 | Nanopore | CS(-) | mREV(-) | 1247047 | 0 | 1178214 |  | 574 |  |
| **200** | S247 | 2024-NGS-3927 | Zika | F | 48 | Y, Thailand | Zika virus | Virus | 34.40 | Nanopore | CS(-) | mREV(-) | 1484571 | 0 | 1121531 |  | 569 |  |

*The patient’s travel history was not available and was recorded as N/A.

**Supplementary Data S3**: Supplementary protocol to article entitled: “Development and performance evaluation of a clinical metagenomics approach for identifying fastidious pathogens in the whole blood from patients with undifferentiated fever”. Data shown is the entire protocol that was developed and followed throughout the study.

# SAMPLE SISPA PROCESSING

# CLINICAL METAGENOMICS PIPELINE FOR CLINICAL SAMPLES

# Protocol

## Nucleic acid extraction

1. Centrifuge EDTA blood tube at 2500 rcf for 5 minutes.
2. Extract nucleic acid from whole blood and plasma into separate tubes
3. Freeze the remaining sample at -80 °C.

## PLASMA ONLY: DNase treatment – TURBO™ DNase

1. Set heat block to 37 °C.
2. Prepare the following reagents to be added to sample isolates:

| Reagent | Volume (1rxn) |
| --- | --- |
| 10x TURBO™ DNase buffer | 1.5 μl |
| TURBO™ DNase | 1 μl |
| NA isolate | 15 μl |
| Total | 17.5 μl |

1. Add 2.5 μl of DNase master mix to each sample isolate.
2. Incubate reaction mixture at 37 °C for 30 min.
3. Add 2 μl of inactivation buffer to each sample.
4. Incubate for 5 min at room temperature.
5. Transfer 10 μl to new tube. Take care not to transfer beads.

## PLASMA ONLY: QIAseq FastSelect rRNA/Globin depletion

1. Prepare the following reagents to be added to DNase treated plasma sample isolates:

| REAGENT | Volume (1rxn) |
| --- | --- |
| FastSelect -rRNA HMR | 0.1 μl |
| FastSelect -Globin | 0.1 μl |
| H_2_0 | 1.8 μl |
| 5x SSIV Buffer | 3 μl |
| DNase treated RNA | 10 μl |
| Total | **15 μl** |

1. Add 5 μl of prepared master mix to DNase treated plasma sample isolates.
2. Run QIAseq FastSelect stepdown PCR:

94°C for 30s

stepdown -5°C/step for 2min → 75 to 55°C

37°C for 2min

25°C for 2min

4°C hold

## BOTH: SISPA-A (cDNA synthesis – SuperScript™ IV)

1. Prepare the following reagents for both sample types:

| First strand primer MMX reaction | | |
| --- | --- | --- |
|  | **Plasma** | **Whole blood** |
| Reagent | **Volume (1rxn)** | **Volume (1rxn)** |
| 250 μM SISPA-A | 1 μl | 1 μl |
| 10 mM dNTP Mix | 1 μl | 1 μl |
| Sample RNA | 15 μl | 10 μl |
| Total | **17 μl** | **12 μl** |

1. Add 2 μl of first strand primer master mix to each sample.
2. Mix and spin down.
3. Incubate at 65 °C for 5 minutes then snap cool on cold block.
4. Prepare the first strand master mix separately. One for each sample type:

| First strand synthesis MMX reaction | | |
| --- | --- | --- |
|  | **Plasma** | **Whole blood** |
| Reagent | **Volume (1rxn)** | **Volume (1rxn)** |
| 5x SSIV Buffer | 1 μl | 4 μl |
| 100mM DTT | 1 μl | 1 μl |
| RNase inhibitor | 1 μl | 1 μl |
| SuperScript™ IV RT | 1 μl | 1 μl |
| Primer MMX rxn | 17 μl | 12 μl |
| Total | **21** **μl** | **19 μl** |

1. Add 4 μl of correct master mix to the plasma and 7 μl of correct master mix to whole blood. Note the different volumes of master mix and make sure the correct one is being used.
2. Mix and spin down.
3. Start the following program on a thermocycler:

23°C for 10min

53°C for 10min

80°C for 10min

1. Prepare the following second strand primer master mix for both sample types:

| Second strand primer MMX reaction | | |
| --- | --- | --- |
|  | **Plasma** | **Whole blood** |
| Reagent | **Volume (1rxn)** | **Volume (1rxn)** |
| 1x Klenow react. buff. | 2.5 μl | 2.5 μl |
| 10 μM SISPA-A | 1 μl | 1 μl |
| 10 μM dNTP | 1 μl | 1 μl |
| First strand cDNA | 21 μl | 19 μl |
| Total | **25.5** **μl** | **23.5** **μl** |

1. Add 4.5 μl of master mix to each well.
2. Mix and spin down.
3. Incubate at 95°C for 3min, cool to room temperature.
4. Prepare 1 μl of Klenow fragment for each sample of plasma and whole blood and add to mixture.
5. Start the following program on a thermocycler:

37°C for 1h

4°C hold

## SISPA-A cDNA Clean up

1. Following second strand synthesis using Klenow fragment clean the samples.
2. Add 1:1 volume of AMPureXP magnetic beads and incubate on a mixer for 5 min at 800 rpm.
3. Make sure the beads are resuspended.
4. Prepare 400 μl of fresh 80 % ethanol per sample.
5. Collect beads on magnetic stand.
6. Remove supernatant.
7. Wash twice with 200 μl of 80 % ethanol, do not resuspend beads.
8. After removing the second ethanol wash make sure to remove all the ethanol.
9. Dry beads until they lose their sheen (make sure to not over dry them).
10. Add 32.5 μl of laboratory grade H_2_O.
11. Incubate on a mixer for 5 min at 800 rpm and make sure the beads are resuspended.
12. Place on magnetic stand.
13. Transfer 30 μl of supernatant to a new tube. Take care not to transfer magnetic beads.

## PLASMA ONLY: SISPA-B (amplification from clean SISPA-A cDNA)

1. Prepare the following master mix:

| PLASMA: SISPA-B | |
| --- | --- |
| Component | **Volume (1rxn)** |
| H_2_O | 16.25 μl |
| 5x Q5 reaction buffer | 5 μl |
| Q5 Hot Start Polymerase | 0.25 μl |
| 10 mM dNTPs | 0.5 μl |
| 100 μM SISPA-B | 0.5 μl |
| Plasma SISPA-A cDNA | 2.5 μl |
| Total | **25 μl** |

1. Add 22.5 μl of master mix to 2.5 μl of clean plasma SISPA-A cDNA.
2. Start the following program on a thermocycler:

98°C for 30s

30 cycles:

98°C for 10s

54°C for 30s

72°C for 1min

72°C for 10min

4°C hold

## PLASMA ONLY: SISPA-B Clean up

1. Following second strand synthesis using Klenow fragment clean the samples.
2. Add 1:1 volume of AMPureXP magnetic beads and incubate on a mixer for 5 min at 800 rpm.
3. Make sure the beads are resuspended.
4. Prepare 400 μl of fresh 80 % ethanol per sample.
5. Collect beads on magnetic stand.
6. Remove supernatant.
7. Wash twice with 200 μl of 80 % ethanol, do not resuspend beads.
8. After removing the second ethanol wash make sure to remove all the ethanol.
9. Dry beads until they lose their sheen (make sure to not over dry them).
10. Add 32.5 μl of laboratory grade H_2_O.
11. Incubate on a mixer for 5 min at 800 rpm and make sure the beads are resuspended.
12. Place on magnetic stand.
13. Transfer 30 μl of supernatant to a new tube. Take care not to transfer magnetic beads.
